# Continuous orientated growth of scaled single-crystal 2D monolayer films

**DOI:** 10.1039/d1na00545f

**Published:** 2021-10-29

**Authors:** Ziyi Han, Lin Li, Fei Jiao, Gui Yu, Zhongming Wei, Dechao Geng, Wenping Hu

**Affiliations:** Tianjin Key Laboratory of Molecular Optoelectronic Sciences, Department of Chemistry, School of Science, Tianjin University and Collaborative Innovation Center of Chemical Science and Engineering Tianjin 300072 P. R. China gengdechao_1987@tju.edu.cn; Institute of Molecular Plus Tianjin 300072 P. R. China linli2020@tju.edu.cn; Beijing National Laboratory for Molecular Sciences, Organic Solid Laboratory, Institute of Chemistry, Chinese Academy of Sciences Beijing 100190 P. R. China; State Key Laboratory of Superlattices and Microstructures, Institute of Semiconductors, Chinese Academy of Sciences Beijing 100083 China

## Abstract

Single-crystal 2D materials have attracted a boom of scientific and technological activities. Recently, chemical vapor deposition (CVD) shows great promise for the synthesis of high-quality 2D materials owing to high controllability, high scalability and ultra-low cost. Two types of strategies have been developed: one is single-seed method, which focuses on the ultimate control of the density of nucleation into only one nucleus and the other is a multi-seed approach, which concentrates on the precise engineering of orientation of nuclei into a uniform alignment. Currently, the latter is recognized as a more effective method to meet the demand of industrial production, whereas the oriented domains can seamlessly merge into a continuous single-crystal film in a short time. In this review, we present the detailed cases of growing the representative monocrystalline 2D materials *via* the single-seed CVD method as well as show its advantages and disadvantages in shaping 2D materials. Then, other typical 2D materials (including graphene, *h*-BN, and TMDs) are given in terms of the unique feature under the guideline of the multi-seed growth approach. Furthermore, the growth mechanism for the 2D single crystals is presented and the following application in electronics, optics and antioxidation coatings are also discussed. Finally, we outline the current challenges, and a bright development in the future of the continuous orientated growth of scaled 2D crystals should be envisioned.

## Abstract 2

Recent advances in growing scaled 2D single-crystal monolayer films by the chemical vapor deposition method are comprehensively presented.

## Graphical abstract

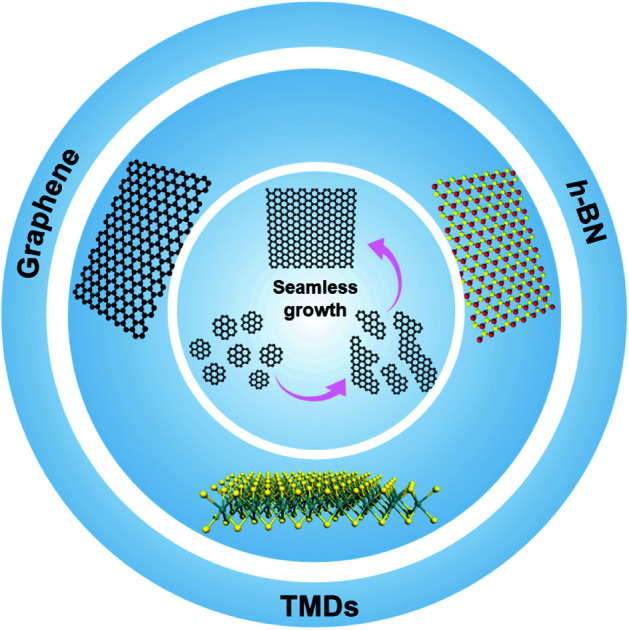

## 1. Introduction

2D materials have emerged as star materials for applications in chemistry, materials science and physics owing to their extraordinary and fascinating property profiles.^1–5^ In the past decades, significant developments have been witnessed in the preparation, characterization and application of 2D materials.^6–10^ Taking the most typical 2D material graphene as an example, great advances with respect to its synthesis method and relevant unique properties have progressed inspiringly since it was first isolated.^6^ On the basis of the development of graphene, it is highly desirable that graphene would revolutionize the industry. However, only materials that meet the requirements of industrial production can occupy the potential material market.^7^ To realize the ultimate goal of 2D materials, fabrication matters a lot and serves as the first step. As Professor Zhongfan Liu from Peking university always said, “fabrication determines future”. Thus, the large-area high-quality production of 2D materials plays a vital role in developing 2D materials and related wide applications.

**Fig. 1:**
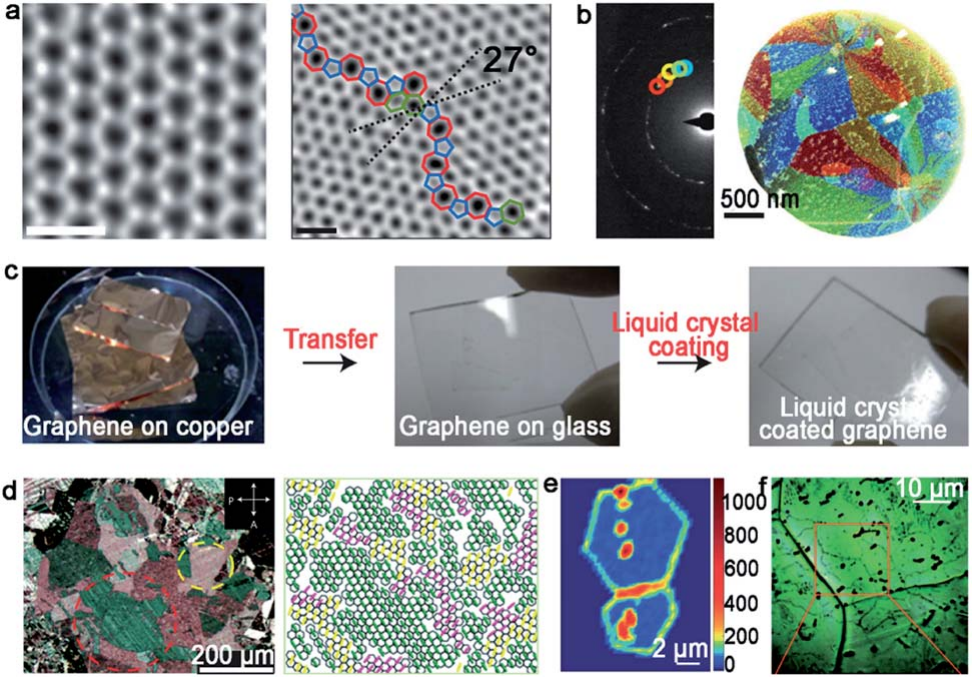
Typical technique means of identifying GBs. (a) The ADF-STEM image showing the defect-free and GBs structure of the graphene grain, respectively. Scale bar: 5 Å. (b) Dark-field image and corresponding maps of the graphene structure. (a and b) Reproduced with permission.^11^ Copyright 2011, Springer Nature. (c) The process of the graphene film coated by nematic liquid crystals. (d) Liquid-crystal molecules with different birefringent colors exhibiting various orientations of graphene grains. (c and d) Reproduced with permission.^15^ Copyright 2012, Springer Nature. (e) Raman intensity mapping of the D band for two joining grains with GBs. Reproduced with permission.^16^ Copyright 2011, Springer Nature. (f) Optical images of the graphene film after oxidation. Reproduced with permission.^17^ Copyright 2012, Springer Nature.

As for 2D materials, single crystals and polycrystals are presented as the two most typical kinds. The significant difference between them is the presence or absence of grain boundaries (GBs). Currently, two obvious GBs are observed. One is the intragranular GBs, which forms through a common nucleation center. The other is called intergranular GBs, and it is formed by independent nucleation sites. In general, nucleation distributes randomly on the metal surface. Thus, GBs inevitably appear when the adjacent domains coalesce together as growth progresses. Taking graphene as an example, GBs usually present a series of pentagons and heptagons or pentagon–heptagon pairs, which are demonstrated by atomic-resolution ADF-STEM (Fig. 1a).^11^ Thereinto, dark-field TEM (DF-TEM) is an effective tool to achieve detection at the nanometer scale. As shown in Fig. 1b, the location, shape and orientation of numerous grains can be distinguished. The map of the graphene structure is formed *via* the diffraction-filtered imaging method. On top of that, TEM and STM techniques are also useful to the identification of GBs at the atomic scale arrangement.^12–14^

It should be noted that the aforementioned methods are mainly utilized to detect local GBs in 2D materials. As for the global information of GB patterns, the nematic liquid-crystal (NLC) method displays relative advantages. It can macroscopically visualize graphene domains *via* polarized optical microscopy (POM) (Fig. 1c).^15^ During this process, the liquid crystal molecule presents different orientations based on the directions of the grains underneath (Fig. 1d). The phenomenon can be ascribed to the interaction between the liquid crystal molecule and graphene. Besides that, Raman spectroscopy is a valid technique to recognize the existence of GBs. Apparently, the strong intensity of the D-band in the Raman mapping confirms the presence of GBs directly (Fig. 1e).^16^ In 2012, Duong *et al.* proposed a method that can directly observe graphene GBs using optical microscopy *via* selectively oxidizing the Cu foil underneath.^17^ UV irradiation is conducted to produce O and OH radicals under the moisture-rich ambient atmosphere, and followed the generated radicals diffusion across graphene GBs. After that, the Cu foil underneath graphene will be oxidized with a larger volume than that used previously. Thus, the visualization of GBs can be achieved by optical microscope (Fig. 1f). Later, Nguyen *et al.* adopted a similar approach to justify the GBs by combining macroscale microscopy images and UV-treatment.^18^

Unquestionably, GBs play a critical role in the properties, such as electronics, chemical reactivity, and thermology. As reported, a GB region is more reactive in a chemical reaction than the surrounding domains. For the electrical performance, it has been widely reported that GBs can act as primary carrier scattering centers to degrade the large-scale device performance.^19^ Taking advantage of the decreasing electrical potential at the GBs, electrostatic force microscopy (EFM) was also conducted to detect GBs.^20^ Recently, deep insight into the formation and influence of GBs during the growth process has been reviewed.^21^ A comprehensive summary about the dynamic formation of GBs, techniques for characterizing GBs, and variation of the materials' properties are presented.

Currently, the production methods of large size monocrystalline 2D materials still remain immature and expensive. The commonly employed approaches include mechanical exfoliation, reduction of graphene oxide (GO) and chemical vapor deposition (CVD), which are briefly introduced in this part. The 2D material flakes can be synthesized through the exfoliation of the corresponding layered bulk counterparts.^22–25^ The exfoliation method can provide intrinsic 2D material samples with less defects and residues. Therefore, the as-obtained sample is a proper candidate for the exploration of the physical properties of 2D materials. The Lee group proposed the layer-engineered exfoliation approach,^23^ which mainly used different metal depositions to control the spalling depth. The phenomenon was attributed to the different interfacial binding energy between the deposition metal and graphene. This method could obtain large-size 2D materials, including *h*-BN and graphene. However, the exfoliation approach always results in low exfoliation efficiency and the as-produced samples are polycrystalline, which degrades the device performance. Another method that is widely used refers to the oxidation and reduction of 2D materials.^26,27^ For example, graphite oxide is prepared by treatment of oxidizing agents and then exfoliated into individual few-layer or monolayer GO. Finally, the GO is reduced by hydrogen annealing and then leads to the formation of graphene. The as-obtained 2D samples by this method usually contain a high density of defects and a relatively inferior quality. Meanwhile, the layer number within the samples are not uniform with a wide distribution.

**Fig. 2:**
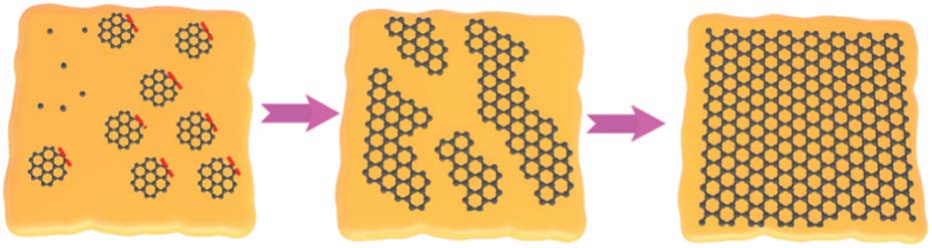
Schematic illustration showing the growth process of large-area single-crystal 2D materials by stitching the domains with a coinciding orientation.

To overcome the above issues, the CVD method has garnered a lot of attention in synthesizing large-area single-crystal 2D materials. For the CVD method, precursors are introduced into the furnace and flow across the hot zone, where the precursors are decomposed into several radicals with the aid of catalysts. Then, the radicals are nucleated and grow into a continuous film. The merits are highly related with its features, such as high controllability, high scalability and ultralow cost. Furthermore, the fine control of the size, morphology, layer number and quality can be realized by this growth process. Strictly, the formation of the 2D materials in CVD follows classical nucleation and growth behavior. Thus, by modulating the CVD conditions, the nucleation and growth of the 2D materials can be precisely tuned. Based on the discussion, the CVD method is the most promising alternative to fabricate large-area single-crystal 2D materials. There are two strategies for growing single-crystal 2D materials in the CVD process. One approach is to suppress the nucleation density to make the nuclei grow as large as possible. This goal can be achieved by processing metal substrates at high temperature, such as annealed copper foil, pre-oxidized copper, or polished copper foil.^28–32^ It is reasonably proposed that the size of the nuclei will expand among the whole surface if the number of nuclei is restricted to only one. However, in this case, the self-limiting mechanism may terminate the growth of large-area 2D materials and the process of covering the entire surface requires a relatively long time. Another approach focuses on precisely controlling the orientations of the domains and ensuring that they stitch into homogeneous 2D materials without GBs (Fig. 2). Compared with the first single-seed method, the multi-seed approach allows for readily preparing large size 2D materials without a sensitive influence on the growth conditions, and without difficulty in precisely keeping the balance between nucleation and growth. With those unique features, great progress has been made in aligning the nuclei of the 2D materials into uniform orientation.^33–35^ Currently, several techniques have been developed to realize the aim, most of which mainly focus on the processing of the catalyst surfaces.^36,37^ It is well acknowledged that the substrate underneath is a critical factor to influence the growth behaviors of the 2D materials because of the existence of the van der Waals interaction. Under this circumstance, a review on this topic is urgently desired to comprehensively present the very recent progresses in the continuous orientated growth of single-crystal 2D materials.

In this review, progress on the continuous orientated growth of large-area single-crystal 2D materials by CVD method are demonstrated. We will first present the detailed cases of growing the representative monocrystalline 2D materials *via* single-seed CVD method, and show its advantages and disadvantages in shaping 2D materials. Then, several typical 2D materials are listed to display the unique multi-seed growth, such as graphene, *h*-BN and TMDs. In this part, it is shown that scaled graphene and *h*-BN crystals are fabricated *via* aligned growth of nuclei on metal substrates. Moreover, several metal catalysts are provided to realize such growth, of which the most intensive attention has been paid to the metal Cu. In the case of the Cu catalyst, it is worth noting that different crystalline forms of Cu might lead to rather remarkable results in the continuous growth. Thus, copper substrates with different Miller index planes will be demonstrated, such as Cu (111), Cu (110) and others. As alternative candidates, Ge and Cu–Ni alloy are presented as well. To elucidate the unique growth at the microscale, theoretical calculations are employed and combined with experiments to probe the detailed mechanism of continuous growth, offering a guideline for the 2D crystal growth. Meanwhile, some characterization means of detecting the single-crystalline and polycrystalline film are presented as well. Furthermore, various applications for the 2D single crystals are being developed, such as electronics, optics and antioxidation coatings. Finally, with continuous efforts devoted, a bright future of the continuous orientated growth of scaled 2D crystals is expected.

## 2. Production methods

Speaking of the growth of 2D single crystals, an essential bottom-up method is referred as CVD. Nowadays, it has already been developed in a perfect way. During this process, defects such as GBs usually inevitably appear. The occurrence of GBs would separate the as-grown samples into individual crystals, which leads to the poor electrical and mechanical functions. As aforementioned, the CVD method can simply achieve the control of the growth conditions. Consequently, it exhibits excellent merits in growing high quality 2D materials. With intensive research in CVD, there are two mainstream ways to solve this problem. One is the single-seed method and the other is the multi-seed method. Meanwhile, the techniques utilized to detect GBs are developed as well. Next, we will fully introduce the two growth strategies by taking the example of graphene growth. The structural characterization of GBs also will be presented by virtue of detailed cases.

### 2.1 Single-seed approach

**Fig. 3:**
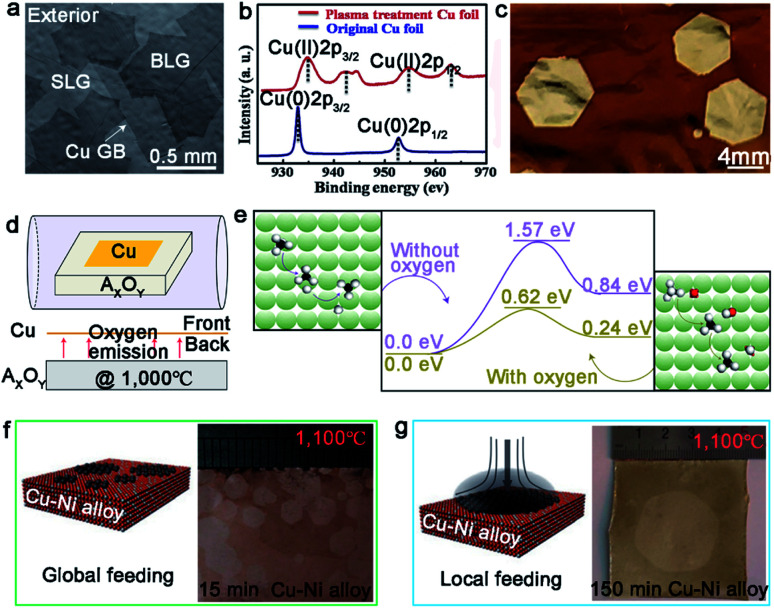
Growth of single-crystal graphene on Cu foil by single-seed method. (a) SEM images of Bernal stacking graphene domains with oxygen. Reproduced with permission.^29^ Copyright 2019, Springer Nature. (b) Cu 2p XPS spectra of the original Cu foil and plasma treated Cu foil. (c) Image of ∼5 mm single-crystal graphene domains after oxidizing Cu foil. (b and c) Reproduced with permission.^31^ Copyright 2019, Elsevier. (d) Schematic showing the special experimental setup with oxide substrate underneath the Cu foil. (e) The decomposition energy of CH_4_ on the Cu (100) surface with and without oxygen, respectively. The green, black, red and white spheres represent Cu, C, H and O atoms, respectively. (d and e) Reproduced with permission.^38^ Copyright 2016, Springer Nature. (f) Schematic illustrations of the global feeding CVD growth method. The left is the optical image of the as-obtained samples. (g) The drawing showing the local feeding growth method on the Cu–Ni alloy. The left is the optical image of 1.5 inches single-crystal graphene. (f and g) Reproduced with permission.^39^ Copyright 2016, Springer Nature.

The single-seed method is a simple and direct approach by substantially reducing the nucleation density, and allowing the limited domains to grow as large as possible. There are two easy ways to do that, and one is modulating the surface characteristics of the metal substrates to control the active sites before the growth. Much effort has been spared to demonstrate that the introduction of the oxygen gas is a good means to attain this goal.^28–32,38^ By blowing the oxygen gas, not only the active sites of the metal surface will be passivated to achieve the goal of controlling the nucleation density, but the domain growth can also be accelerated by reducing the energy barrier of the dissociation of CH_4_ and change the growth kinetics from edge-attachment-limited to diffusion-limited.^28^ Furthermore, this way can be utilized to grow Bernal (AB)-stacked bilayer graphene (Fig. 3a).^29^ The Ruoff group broke the surface-limit process on the Cu foil to realize the growth of bilayer graphene by extra oxygen introduction. The low energy electron diffraction (LEED) characterization was conducted to demonstrate the monocrystalline lattice of both layers. Moreover, the single crystal exhibited excellent electrical quality. In addition to direct oxygen introduction, dealing with Cu foil by plasma treatment is an efficient manner to produce oxygen, and thus reduce the active sites.^31^ The plasma treatment not only removed impurities on the Cu foil, but also produced CuO nanoparticles on its surface. After that, CuO was reduced and produced oxygen after annealing the copper under H_2_ atmosphere (Fig. 3b). By comparison, the nucleation of the graphene domains was extremely suppressed and the growth rate sharply increased. As a result, the single-crystal graphene domains could reach up to the size of 5 mm in a growth time of 2 h (Fig. 3c).

In recent years, it has been widely recognized that putting another substrate under the metal catalysts accelerated the growth rate. Notably, this method can provide continuous oxygen in the whole process. The Liu group developed a novel method to achieve ultrafast growth of graphene on Cu foil.^38^ They placed an oxide substrate under the Cu foil to supply continuous oxygen (Fig. 3d). Combined characterizations and calculations proved that oxygen released from the oxide substrate significantly decreased the decomposition barrier energy (Fig. 3e) and increased the growth rate. Based on this design, monocrystalline graphene with an average size of 0.3 mm was obtained in just 5 s. Inspired by this method, they changed the oxide substrate to the metal fluoride substrate, and found that fluoride acted the same as oxygen in the growth process.^32^ The average growth rate of the graphene domains could reach up to 200 μm s^−1^, which was three times faster than the above mentioned rate.^38^ Moreover, this method not only worked for the growth of graphene, but also for *h*-BN and WS_2_.

The other way is local feeding, which means setting the carbon precursor to a desired position of the metal surface and making the single nucleus grow into a graphene film covering the surface. Otherwise, the nucleation of graphene tends to distribute randomly, as shown in Fig. 3f. Consequently, the local feeding method achieves the control of the nucleation site to avoid undesirable defects. The Xie group has reported the synthesis of a monolayer graphene film by this method with a size of 1.5 inch in just 2.5 hours, which was wafer-scale single-crystalline graphene (Fig. 3g).^39^ It should be noted that the Cu_85_Ni_15_ alloy was chosen as the substrate in this experiment rather than pure copper, aiming to guarantee the rapid growth rate and the control of the layer numbers.

### 2.2 Multi-seed approach

Although the single-seed method is an effective way to reduce the density of the active sites to achieve the growth of monocrystalline 2D materials, it comes with problems of a lower growth rate and longer time. As a result, it is not the most appropriate method to achieve mass production in the future. Since the achievement of seamless stitching was first proved,^18^ intensive research studies have been implemented to grow monocrystalline 2D materials by multi-seed CVD method under the aid of various catalysts. The achievement of seamless stitching is based on the continuous orientated growth of individual grains. The aim of the scaled production of 2D materials stimulates the development in this territory. Up to now, meter-size monocrystalline graphene has been fabricated on single-crystal Cu^36^ and the success further demonstrates that choosing suitable substrates plays a decisive part in the process. The orientation of domains will align along a certain direction on single-crystal substrates owing to the interaction between them. Besides that, the large-size single-crystal *h*-BN film was grown on Cu (110) vicinal surface with the guidance of Cu 〈211〉 steps.^34^ By comparison with the single-seed strategy, the multi-seed method exhibits greater potential for the fabrication of scaled 2D materials, in which growing and merging the neighboring domains can be conducted in such a short time. However, it should be admitted that there are still dilemmas impeding the mass production of 2D materials *via* multi-seed method. For example, the limited size of high-quality substrates strongly affects the size of the as-obtained 2D film, and the relatively complex dealing process to substrates directly leads to the increase of the economic cost. Thus, it is of great significance to prepare large-area single-crystal substrates by simple method. Recently, the review published by the Chen group took a deep exploration in the growing behaviors of single-crystal 2D materials on monocrystalline substrates.^40^ This article comprehensively summarized the merits and demerits of different substrates, and the response to growth mechanisms were presented as well. In this article, following in part 3, we will further discuss the recent advances of growing single-crystal 2D materials by multi-seed CVD method.

## 3. Continuous orientated growth of large-area 2D materials

Currently, the multi-seed method has been a promising strategy for the large-area production of single-crystal 2D materials, and thus taking deep insights into the different catalytic behaviors of substrates is desirable. In this part, we will summarize the recent advance in the growth of single-crystal 2D materials on Cu, Ge and Cu–Ni alloy by multi-seed method. Several typical 2D materials such as graphene, *h*-BN and TMDs are all mentioned to be applied in the continuous growth strategy.

### 3.1 Graphene

Graphene is composed of carbons positioned in a hexagonal shape, which has gained tremendous attention of scientists from various fields. Up to now, graphene has been an important material in academic research due to many of its prominent properties involving electron mobility, thermal conductivity and optical transparency. Thus, the growth of high-quality graphene has brought unprecedented breakthroughs, and we will summarize the progress under different catalysts systematically in the fashion of the orientated multi-seed approach.

#### 3.1.1 Solid Cu catalyst

It is well acknowledged that a suitable substrate is regarded as the important part for CVD growth. This is because different substrate catalytic abilities and carbon solubility will result in distinctly various graphene growth modes. Generally, graphene film grown on Cu tends to be single layer due to the typical self-limiting mechanism.^41^ Moreover, the structure of Cu (111) is suitable as a substrate owing to the matched symmetry, similar lattice constant and strong interactions, along with a certain orientation between them. In detail, Cu (111) has matched symmetry with graphene, so that the orientation of well-aligned graphene domains is not changed by Cu (111). Besides that, the lattice constant of Cu (111) and graphene is 0.256 nm and 0.246 nm, respectively, which is nearly the same.^42^ Based on the discussion, fabricating large size single crystallographic Cu foils or films is a feasible way to facilitate the evolution of graphene.

**Fig. 4:**
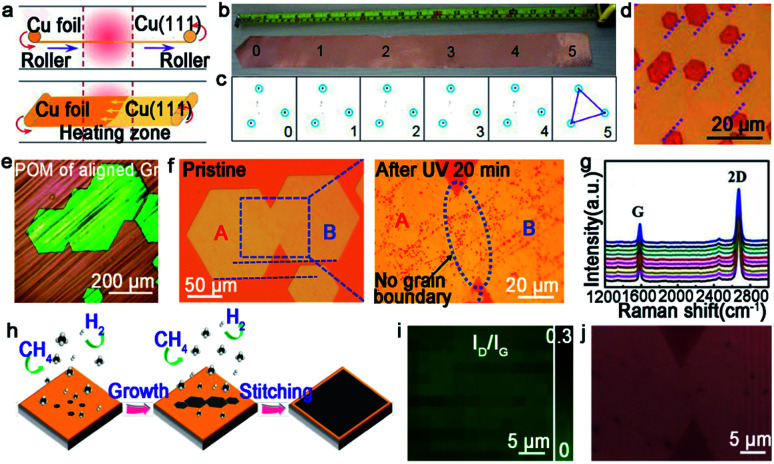
Continuous growth of the large-area single-crystal graphene film on the Cu surface. (a) Schematic showing the temperature-gradient-driven annealing technique. (b) The as-prepared single-crystal Cu (111) foil. (c) Corresponding LEED patterns from the six different regions marked in (b). (d) Optical image of the graphene films after H_2_ etching. The holes indicate the identical direction of the graphene domains. (e) Polarized optical images of aligned graphene. (a–e) Reproduced with permission.^36^ Copyright 2017, Elsevier. (f) Optical microscopy image showing the morphology of the merging region before and after UV-light irradiation. Reproduced with permission.^18^ Copyright 2015, Wiley-VCH. (g) Raman spectra of ten locations of graphene films. (h) Growing process of the graphene film on the Cu (111) film. (i) Raman mapping of *I*_D_/*I*_G_ of the single-crystal graphene film. (j) Optical image of the stitching region of graphene after plasma treatment. (g–j) Reproduced with permission.^44^ Copyright 2019, The Royal Society of Chemistry.

Certainly, great progress of growing single-crystal graphene has been made in recent years. In 2017, the Liu group proclaimed a way of ultrafast growth of a large-area graphene film with the domain alignment of 99% unidirectional orientation.^36^ They adopted a temperature-gradient-driven annealing technique to prepare monocrystalline substrate. The commercial Cu was slowly slid through a central temperature zone, and the temperature gradient provided the main impetus for the movement of GBs on the polycrystal copper (Fig. 4a). As for the mechanism about the formation of the Cu (111) facet, density functional theory (DFT) calculations demonstrated the minimum surface energy of Cu (111) facets among three low index Cu surfaces. Meanwhile, the bond between Cu atoms near the regions of GBs was so loose that GBs could smoothly slide towards the high temperature. As a result, a record size of 5 × 50 cm^2^ single-crystal Cu (111) foil was obtained. Moreover, the crystalline quality of the Cu foil was confirmed by LEED patterns, which have threefold rotation symmetry with coincident rotation angle (Fig. 4b and c). With this as the substrate, the graphene domains began to nucleate under the continuous O_2_ supply and following aligned along the same direction (Fig. 4d). Then, a single-crystal graphene film with the same size with copper was achieved in only 20 min. In order to demonstrate the existence of GBs, they coated the as-obtained graphene film with NLC and further observed *via* POM technique. As shown in Fig. 4e, there were no GBs in the image near the merging regions of the islands. To further measure the quality of the as-grown graphene, it was transferred onto a *h*-BN substrate to fabricate field-effect transistors (FETs). Surprisingly, the mobility could reach up 23 000 cm^2^ V^−1^ s^−1^ at 4 K. In conclusion, this method indeed inspires researchers to become deeply involved in the large-scale production of 2D materials.

For the seamless merging of graphene islands, Nguyen and co-workers achieved continuous film without GBs.^18^ They also proposed that the concept of “seamless” means the process that domains with a coinciding orientation stitch into a continuous film without the presence of GBs. They processed the Cu (111) surface by chemical-mechanical polishing commercial copper repeatedly and annealing it at 1075 °C. As for the growth of graphene, CH_4_ and H_2_ with a high ratio were introduced into the furnace. Then, the hexagonal graphene domains with unidirectional orientation occurred and eventually merged into a continuous film free of GBs. Combining optical microscopy and UV-light irradiated measurements, they proved the seamless stitching between two orientated graphene domains (Fig. 4f). In addition, scientists found a straightforward approach to achieve this just by annealing the Cu foil for a long time at a temperature of 1030 °C in an Ar/H_2_ environment.^43^ After that, precisely controlling the growth parameters led to obtaining a continuous graphene film. This success mainly depended on two key steps, one is the Cu foil recrystallization and the other is optimization of growth parameters. Recently, a single-crystal graphene film with a size of 2 inches was obtained by stitching domains on the Cu (111) film.^44^ The Cu foil was annealed at 1085 °C, a temperature higher than Cu melting point. Under this case, Cu foil would transform into a quasi-atomically smooth Cu film laying on the Mo foil surface. Meanwhile, polycrystalline Cu was transformed into a single-crystal Cu (111) film through atom rearrangement (Fig. 4h). Based on these conditions, the oriented graphene domains grew on the surface and seamlessly coalesced into a continuous graphene film. Conducting a Raman test on the as-grown film, the intensities of the D peaks and the value of *I*_D_/*I*_G_ seemed weak (Fig. 4i). All of the results further confirmed the uniformity and high quality of the graphene film. The defect-free property was also evidenced by plasma treatment result, which was the existence of no GBs in Fig. 4j. Back-gated FETs fabricated based on it offered outstanding mobility of ∼11 500 cm^2^ V^−1^ s^−1^. This new strategy provides the possibility for the scaled production and industrial-level application.

**Fig. 5:**
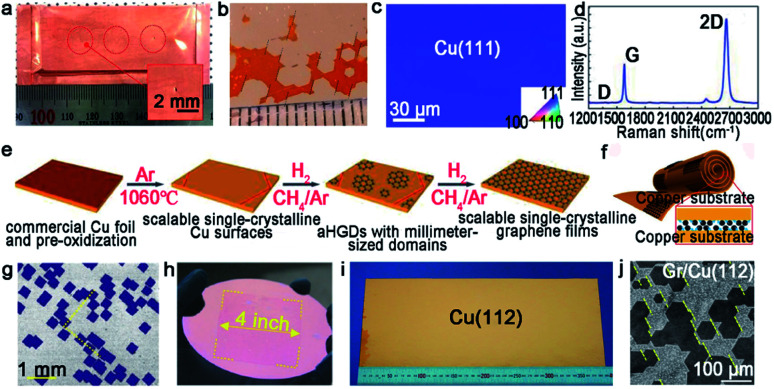
Growing single-crystal graphene on the Cu surface. (a) Photograph of the hole-pocket Cu foil. (b) The as-grown graphene domains with the same orientation. (a and b) Reproduced with permission.^45^ Copyright 2016, The Royal Society of Chemistry. (c) The EBSD image of the Cu (111) surface. (d) Raman spectra of the as-obtained sample. (e) The growth procedure of graphene on the commercial Cu foil. (c–e) Reproduced with permission.^47^ Copyright 2018, The Royal Society of Chemistry. (f) Schematic showing the ultrafast growing graphene arrays on the stacking structure of Cu foils. Inset: schematic of the H_2_/CH_4_ transport and decomposition. (g) Graphene arrays with unidirectional orientation transferred onto the Si/SiO_2_ substrates. (h) Photograph of 4 inches wafer-scale graphene film. (f–h) Reproduced with permission.^37^ Copyright 2016, Wiley-VCH. (i) Single-crystal Cu (112) foil. (j) Graphene domains on the Cu (112) surface with uniform orientation. (i and j) Reproduced with permission.^49^ Copyright 2020, Springer Nature.

As aforementioned, fabricating a large-area single-crystal Cu foil or film is a challenge for graphene industrial production. Because in several cases, dealing with commercial copper is complicated and time-consuming. To solve this problem, Lee *et al.* proposed a hole-pocket method, which could transform commercial polycrystal Cu foil to single-crystal Cu (111) surface spontaneously without any additional treatment of the substrate.^45^ Initially, the Cu foil was made into a rectangular shape, followed by making several holes with a diameter of 0.3–0.4 mm (Fig. 5a). This special structure ensured the constant Cu evaporation, redeposition rates and stable gas flow inside the Cu pocket. Then, the inner surface of the Cu foil rearranged into a thermodynamically stable Cu (111) facet. The behaviors of the graphene crystals are the same as that for the previously reported behaviors, and they grew into hexagonal grains with an accordant spatial orientation. After that, the grains joined into a single-crystal film, covering the whole surface (Fig. 5b). Liquid crystal analysis indicated alignment crystal orientations. Moreover, it is worth mentioning that the back-gated FETs fabricated with the film had a higher mobility of about 17 000 cm^2^ V^−1^ s^−1^ than that of the previous report.^46^ Another simple method has also been proposed by Wang,^47^ whereas the single-crystalline graphene domains were successfully obtained on the commercial Cu. They pre-introduced an oxide layer on the commercial copper, and annealed it under an Ar atmosphere (Fig. 5e). Then, the Cu (111) surface would occur on top of the commercial copper foil (Fig. 5c). As for the growth of graphene, the domains displayed a well-defined alignment with a misorientation angle of only less than 1°. The Raman spectra exhibited a negligible D band, which was an indication of the high quality of the as-obtained samples (Fig. 5d). The same results were observed in other types of commercial Cu foils, and thus indicated that the oxygen-assisted surface mono-crystallization could be applied in a wide range of processing Cu. It may serve as another way to fabricate scaled graphene materials for wide application. However, in fact, there will be additional layers in a small fraction of the large-area single-crystal monolayer graphene film, which lead to the degradation of the electrical transport. Thus, more attention has been paid to probe the formation mechanism of the adlayers and the strategies to eliminate them.^46,48^ Scientists found that the amount of subsurface carbon in Cu foils gave rise to adlayer growth to some extent, and annealing it in hydrogen gas before growing graphene domains would remove the subsurface carbon.^46^ Using this method, a higher quality single-crystal graphene film without adlayer was achieved on Cu (111).

Except for the Cu (111) surface, the Cu foil with different Miller index planes have also been explored in making single-crystal graphene.^37,49,50^ It is a pity that graphene domains growing on the Cu (100) have two predominate orientations, which further shows the mismatched lattice symmetry between graphene and Cu (100).^50^ Meanwhile, this phenomenon does not represent the impossibility of single-crystal graphene growing on the Cu (100) facet. In 2016, the Liu group developed a method to do that with a record rapid growth rate up to 300 μm min^−1^.^37^ It is worth noting that the Cu (100) crystal was obtained by oxygen chemisorption-induced reconstruction with a simple stacking structure (Fig. 5f). During the process, oxygen was introduced by chemisorption in the pre-oxidation process, and enabled the reconstruction of “Cu (100)–(2 × 2)-O” of the Cu foil. Eventually, the Cu (100) single-crystal surface would be obtained by hydrogen reduction to remove oxygen. As for the growth of graphene, 100% square graphene domains aligned along the atomic step on the Cu (100) surface and wafer-scale continuous graphene film could be obtained with the prolonging of the growth time (Fig. 5g and h). Besides that, high-index facets can serve as substrates due to their high catalytic and relatively strong interactions with 2D materials. A recent study reported the fabrication of high-index foils such as Cu (112) foils with the size as ∼39 × 21 cm^2^ (Fig. 5i),^49^ Cu (133), Cu (223) and others. Meanwhile, as revealed by experimental results, the epitaxial growth of graphene (Fig. 5j) and *h*-BN on the high-index facets were truly realized. It is reasonably concluded that this result gives new insight into the growth of 2D materials on the high-index Cu facets. However, the processing of large-area Cu foils with high-index facets normally needs high temperature owing to their higher surface energy, and is thus still challenging for scaled production.

#### 3.1.2 Liquid Cu catalyst

**Fig. 6:**
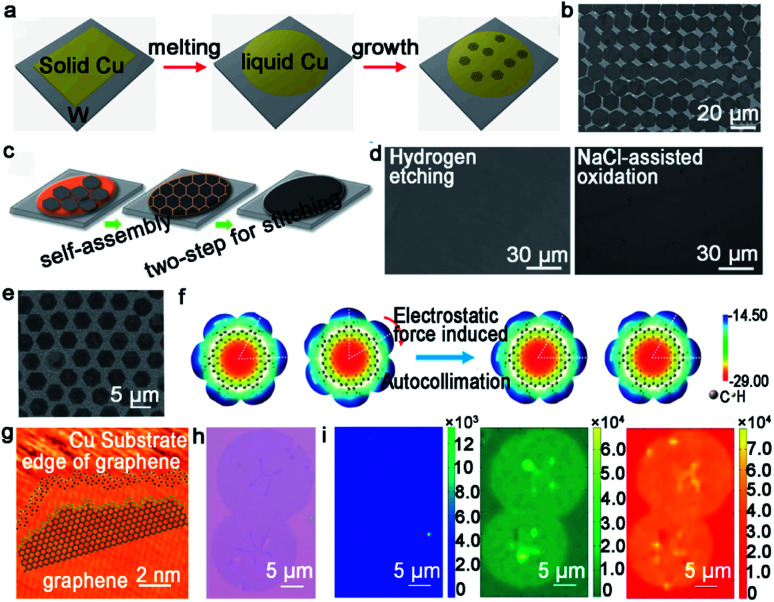
Growth of graphene arrays on liquid Cu. (a) Schematic showing the growing process of graphene arrays on liquid Cu. Reproduced with permission.^51^ Copyright 2012, National Academy of Sciences, USA. (b) Single-crystal graphene arrays under a flow rate of CH_4_ at 5 sccm. Reproduced with permission.^53^ Copyright 2013, WILEY-VCH. (c) Schematic illustration of the two-step stitching growth of graphene. (d) Optical microscope image of the graphene film after hydrogen etching and NaCl-assisted oxidation process. (c and d) Reproduced with permission.^55^ Copyright 2015, American Chemical Society. (e) SEM image of GSOS. (f) Electrostatic potential distribution on the molecular surface. (e and f) Reproduced with permission.^56^ Copyright 2016, American Chemical Society. (g) Atom configuration at the edges of IGG. (h) Optical image of IGGs. (i) Raman mapping of D, G, and 2D peaks of two adjacent IGGs. (g–i) Reproduced with permission.^57^ Copyright 2016, American Chemical Society.

Since the first report on the introduction of liquid Cu as an ideal substrate to grow uniform monolayer single-crystal graphene film by CVD (Fig. 6a),^51^ many scientists have been set about employing liquid metal as a substrate to grow 2D materials. Unlike the solid Cu, the liquid Cu substrate, which is obtained by heating solid copper over the melting point in the reactor, has its own compelling advantages. On the one hand, the size of the domains is easy to be controlled only by tuning the growth parameters. This is in sharp contrast with solid copper, which needs further complex treatments. On the other hand, the domains on the liquid Cu obey the self-assembly mechanism; namely, each domain is prone to assemble along the same orientation and merges into a continuous film. Meanwhile, the surface steps and GBs will be significantly eliminated on the liquid copper surface. Besides that, the Fu group gained deeper insight into the difference of growing graphene on solid Cu and molten Cu substrate.^52^ Not only is the nucleation rate on the molten state substrate accelerated, which is attributed to the rich free electrons of the rheological surface, but the initial nucleation density is also suppressed as a consequence of the isotropic and smooth surface. In brief, the rheological surface of the liquid copper is an excellent platform for the multiple behaviors of the graphene domains.

Based on the previous discovery, the Liu group further employed this intriguing method to grow graphene arrays. In 2014, the precise control of self-aligned single-crystal graphene flakes on the liquid copper foil was realized.^53^ The most fascinating part of this process is centered on the control of the growth parameters. The prefect graphene arrays with uniform size were achieved by precisely controlling the flow rate of CH_4_ gas at 5 sccm (Fig. 6b). Whereas, increasing the CH_4_ flow rate to an optimized value could contribute to the synthesis of bilayer graphene film. In order to further characterize the quality of the crystal, FET devices were fabricated and demonstrated an average mobility of up to 2000 cm^2^ V^−1^ s^−1^. In fact, cracks may be very likely to appear during the cooling process, which can lead to performance degradation. A novel two-stage CVD growth method on melted Cu has been utilized in order to fill the macroscopical defects that formed upon cooling.^54^ Different from the aforementioned reports, the authors chose metal W as the supporting substrate rather than Mo to ensure the continuity of graphene. During the cooling process, the temperature was reduced slowly from 1090 °C to 1060 °C, and simultaneously continued to keep the flow rate of CH_4_ gas so that the cracks achieved regrowth. Eventually, the film was transferred onto the target substrate *via* stress-free wet transfer method. In view of this work, the Kim group employed a new two-step growth process (Fig. 6c).^55^ A low carbon flux was introduced at the initial step to form a normal single-layer graphene film and a relatively high carbon flux was introduced to fill the gaps at the final stage. After that, they conducted hydrogen etching and NaCl-assisted oxidation method, aiming to demonstrate the continuity of the graphene film. As shown in Fig. 6d, both images showed that there were no GBs between the adjacent graphene domains. Meanwhile, a lower electrical resistance of the as-obtained films also clearly proved its continuity.

The Fu group from Wuhan university has made great progress in growing graphene on liquid Cu. For example, they demonstrated for the first time the synthesis of a two-dimensional super-ordered structure on melted Cu.^56^ Taking the graphene super-ordered structure (GSOS) as an example, the mechanism has been fully elaborated. PMMA was chosen as the carbon source and spin-coated onto the Cu surface. Then, the film would decompose and condense on the melting copper to form nucleation seeds with the temperature rising over the melting point of Cu at the hydrogen gas atmosphere. Afterwards, an airflow with uniform speed and direction was blown with the aim to render the even distribution of seeds (Fig. 6e), which was the most energetically favorable state. After that, introducing CH_4_ to promote the growth of seeds and following GSOS would form naturally. Each graphene single crystals in GSOS was rotated to match each other, and all of them over a large area exhibited high orientation consistency. The calculated electrostatic potential maps and molecular structure demonstrated the importance of the electrostatic interaction in forming the as-grown graphene domains (Fig. 6f). The other success is the growth of the isotropic graphene grain (IGG), which is also achieved on the amorphous surface.^57^ The single-crystal IGG was obtained on the liquid Cu surface with mixed edge structure, which resulted in the high activity of the edges (Fig. 6g). Thus, each IGG could just rotate at a low angle to self-adjust their orientation to achieve smooth stitching with the adjacent grains (Fig. 6h). As seen in Fig. 6i, Raman mappings were conducted to affirm the presence of GBs. The intensity of the D bands was very weak over the whole stitching regions, which further demonstrated the smooth stitching of the neighboring grains. The intensity of G and 2D Raman peaks confirmed the uniformity of the stitching regions. It is straightforward that the rheological surface, without any certain introduction of grain orientation or a mainstream growth rate toward a defined direction, provides the possibility to grow round IGG. Meanwhile, this method provides a guide for the seamless growth of 2D single-crystals on the liquid metal.

#### 3.1.3 Ge catalyst

**Fig. 7:**
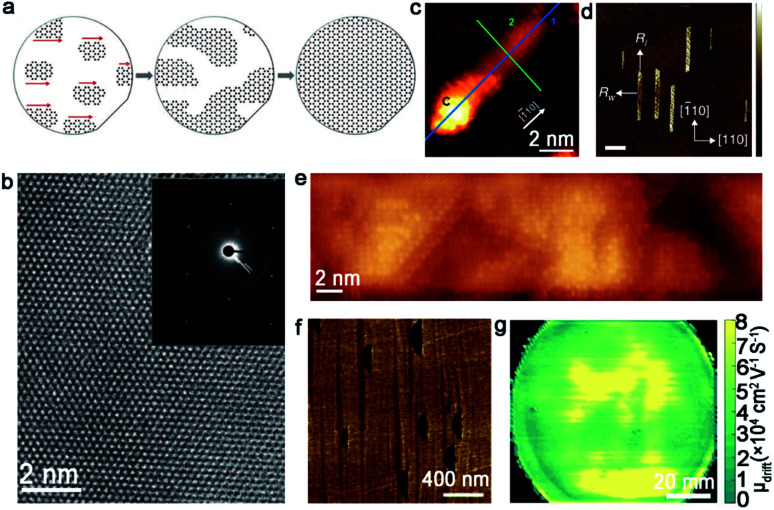
Growth of single-crystal graphene on the Ge substrate. (a) Schematic of growing the single-crystal monolayer graphene on the Ge (110) surface. (b) High-resolution TEM image of the graphene film. Inset: SAED image of the single-crystal graphene. (a and b) Reproduced with permission.^33^ Copyright 2014, American Association for the Advancement of Science. (c) STM image of GNR with a C cluster at its end. Reproduced with permission.^58^ Copyright 2020, Elsevier. (d and e) AFM and STM image of GNRs on the Ge (001) surface. Scale bar is 400 nm in d. Reproduced with permission.^60^ Copyright 2015, Nature Publishing Group. (f) Graphene islands on the Ge (001) substrate with a 15° miscut angle. (g) Terahertz wafer-scale mobility mapping of the graphene film on the vicinal Ge (001) substrate. (f and g) Reproduced with permission.^63^ Copyright 2020, Elsevier.

The metal Ge has also been found to be a good candidate for the continuous growth of 2D materials because of its catalytic activity and low carbon solubility. Thereinto, a Ge (110) surface with two-fold symmetry is introduced into growing a uniform 2D single-crystal film. In 2014, researchers developed a novel method to produce graphene by using the H-terminated Ge (110) substrate.^33^ The graphene islands with an identical orientation were aligned along the [−110] direction of the substrate, and subsequently merged into a continuous monolayer graphene *via* low-pressure CVD (Fig. 7a). The as-produced graphene film was high quality without any visible defects and exhibited a perfect single-crystal lattice, which was demonstrated by selected area electron diffraction (SAED) images (Fig. 7b). It should be noted that the anisotropic Ge–C covalent bond and high rotation energy ensured the identical orientation of the graphene domains. Furthermore, the as-obtained graphene film was directly mechanically exfoliated from the substrate underneath, instead of etching the substrate with chemical etchants. The utilization of the etch-free dry transfer method could be ascribed to the weak adhesion existing between Ge and the graphene film. This success triggered widespread attention in the study about the growth of graphene on the Ge (110) substrates. Based on this study, the Kim group stepped forward for the exploration of graphene behavior on the Ge (110) facet.^58^ A large-area graphene film was obtained by flowing ethylene precursor gases into the low-pressure CVD system at 700–800 °C. Different from the abovementioned work, as an intermediate phase, C cluster seeds were found on the Ge (110) terraces in the initial stage of graphene nanoribbons (GNRs) formation. The GNRs were aligned along the [−110] direction, which was consistent with a previous report (Fig. 7c). Then, the GNRs transformed into graphene nanoislands and eventually merged into a monocrystalline monolayer graphene film. During the growth process, optimizing the growth parameters (such as the CH_4_/H_2_ ratio) is also important. Wang *et al.*^59^ achieved 4 inches of the monolayer single-crystal graphene on the Ge (110) substrate *via* choosing a lower CH_4_/H_2_ ratio. This work may inspire scientists to better optimize the growth conditions to fabricate large-area high-quality graphene on the Ge substrate.

Unlike the graphene domains epitaxial growth on the Ge (110) surface, the crystals on Ge (001) are prone to expand into semiconducting nanoribbons with a high aspect ratio and smooth armchair edges. It was reported that the oriented growth of the armchair GNRs was achieved on Ge (001), and the nanoribbons were self-aligned along the Ge 〈110〉 direction.^60^ Thus, two ribbon orientations were perpendicularly aligned with equal probability as a consequence of the growth of highly anisotropic GNRs (Fig. 7d and e). To ensure all of the graphene nanoribbons aligned along one orientation instead of two perpendicular directions, vicinal Ge (001) was employed to address this issue. Jacobberger *et al.* realized the ∼90% alignment of nanoribbons by tuning the miscut angle.^61^ Based on these studies, the Di group achieved direct growth of unidirectional GNRs, with over 98% well aligned on Ge (001) surface by designing a miscut of 12°.^62^ Aiming to achieve 100% single direction alignment of the graphene domains, the authors further increased the miscut angle to 15°.^63^ In this case, all of the graphene domains were perpendicular to the miscut direction, and seamlessly stitched into a wafer-scale graphene film as the growth progressed (Fig. 7f). The as-obtained graphene film exhibited ultrahigh carrier mobility of about 2 × 10^4^ cm^2^ V^−1^ s^−1^ (Fig. 7g), which paved the way to manufacture graphene-based nanoelectronic devices with higher performance. The unique and detailed mechanism will be explained in section 4.

#### 3.1.4 Other substrates

**Fig. 8:**
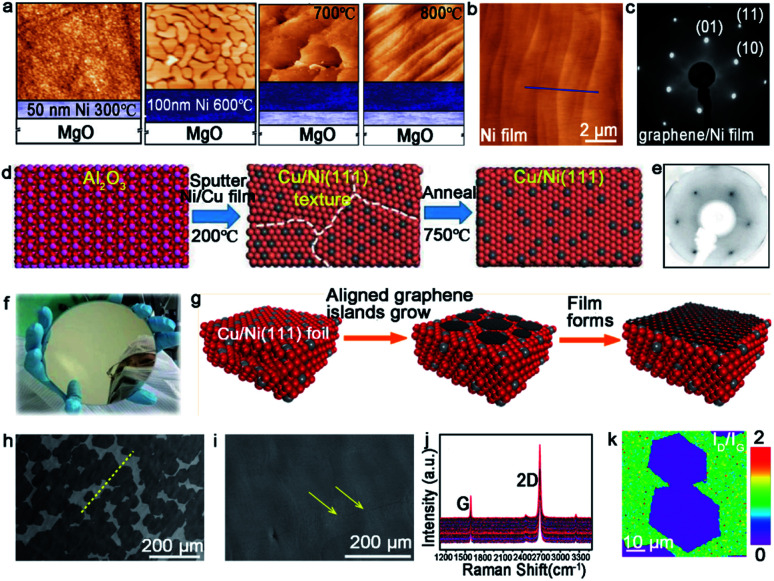
Growth of the large-area single-crystal graphene film on other substrates. (a) Productive process of the single-crystal Ni (111) film. (b) AFM image of the as-obtained Ni film. (c) LEED pattern of graphene grown on a Ni film. (a–c) Reproduced with permission.^68^ Copyright 2011, American Chemical Society. (d) The fabrication process of the single-crystal Cu/Ni (111) film. (e) LEED pattern of graphene. (f) Photograph of the as-obtained 6 inches Cu/Ni film. (d–f) Reproduced with permission.^64^ Copyright 2019, WILEY-VCH. (g) Illustration showing the stitching of the graphene islands on the Cu/Ni (111) alloy. (h) SEM images of the graphene domains. The yellow dash line presents the direction of domains. (i) Continuous graphene film derived from the stitching of the domains in (f). (j) Raman spectra of numerous regions taken from the graphene film. (k) Raman mapping of the *I*_D_/*I*_G_ intensity ratio of the joined islands. (h–k) Reproduced with permission.^70^ Copyright 2018, American Chemical Society.

Other types of substrates^64–66^ have been adopted to grow monocrystalline graphene in recent years. Among the transition metals, Ni has been intensively studied for the growth of high-quality graphene.^65,67^ Ni possesses different growth mechanisms with Cu due to the difference of the carbon solubility. The graphene film grown on the Ni surface prefers to be a multilayer. Thus, thin Ni foil has been widely employed to achieve the growth of multilayer graphene.^41,67^ The Starke group reported a novel method for the growth of single-crystal graphene on a single-crystal Ni (111) film.^68^ The detailed process of the Ni (111) film is shown in Fig. 8a. A 50 nm thick Ni buffer layer was first deposited on the MgO surface at 300 °C, and subsequently covered with a 100 nm thick Ni film by deposition at 600 °C. After depositing the Ni films on the MgO surface, annealing the whole substrate at 700–800 °C in an ultrahigh vacuum (UHV) system allowed for the attainment of a continuous Ni film without GBs. The AFM image exhibited the periodic structure of terraces and steps on the Ni film (Fig. 8b). In this case, propylene gas was introduced to realize the preparation of monolayer graphene. The LEED patterns of graphene on the Ni film demonstrated that the growth of graphene followed the directions of the monocrystalline substrate (Fig. 8c).

Apart from the Ni catalyst, the Cu–Ni alloy has attracted much attention in growing a high quality graphene film as well. Evidence shows that the doped Ni atoms on the Cu–Ni alloy not only maintains its own high catalytic capacity, but also improves the activity of its adjacent Cu atoms.^69^ Zhang and co-workers realized the growth of the wafer-scale single-crystal graphene film on the Cu/Ni (111)/sapphire wafers at a low temperature.^64^ During this process, the Cu/Ni (111) single-crystal thin film was fabricated by sputtering the Cu_85_Ni_15_ alloy target onto an α-Al_2_O_3_ (0001) wafer at 200 °C, and following annealing at 750 °C under an Ar/H_2_ atmosphere (Fig. 8d). The excellent catalytic ability of the Ni atoms provided a platform for the growth of graphene at a low temperature. When nucleation and growth occurred, the individual graphene domains coalesced perfectly into a continuous film (Fig. 8f). The LEED images showed only one set of diffraction patterns, which confirmed the single-crystal structure of the whole graphene film (Fig. 8e). The Ruoff group succeeded in achieving the ultra-fast growth of the monocrystalline graphene film on the Cu/Ni (111) alloy.^70^ The most intriguing part is the fabrication of the single-crystal substrate. The Cu (111) foil was first fabricated as the original material for the next step. Then, the Ni layers were electroplated on the two sides of the Cu (111) foil, and heated at 1050 °C to obtain the ordered structure of the Cu and Ni atoms (Fig. 8g). Using this alloy as a catalyst, the precursors were introduced into a reactor at 1075 °C and grew into graphene domains with the identical orientation (Fig. 8h). The full coverage of the monolayer graphene film was realized only in 5 min (Fig. 8i). The weak D and *I*_D_/*I*_G_ intensity peak between the joined islands in the Raman mapping demonstrated the absence of obvious GBs (Fig. 8j and k).

To better understand the aforementioned works, two tables have been provided to summarize the recent advances. Table 1 summarizes the detailed growing parameters and performance of single-crystal graphene. Meanwhile, Table 2 presents the dealing process for different metal catalysts with respect to Table 1.

**Table 1:** Summary of the detailed growth parameters and performance of single-crystal graphene

Gas flow (sccm)	Press.	Temp. (°C)	Time (min)	Alignment	Size	Performance		Ref.
						Electrical mobility (cm^2^ V^−1^ s^−1^)	Sheet resistance (Ω sq^−1^)	
CH_4_: 3 H_2_: 50 H_2_/CH_4_: 1600 Ar: 1000	AP	1075	60	98%	6 × 3 cm ^2^	6100 (average)	__	18
CH_4_: 1–5 H_2_: 500 Ar: 100	AP	1030	20	>99%	5 × 50 cm ^2^	23 000 (4 K) 15 000 (RT)	230	36
CH_4_: 6 H_2_ Ar	26 Torr	1030	90	95%	__	__	__	43
CH_4_: 2–3 H_2_: 100 Ar: 300	AP	1070	__	99%	2 inches	11 500	315	44
CH_4_: 15 H_2_: 25 Ar: 500	AP	1050	360	100%	__	17 000	__	45
CH_4_: 20 H_2_: 10 Ar: 300	AP	1060	40	98%	Centimeter scale	__	270 ± 30	47
CH_4_: 0.5–2.0 H_2_: 100–200	2 Torr	1060	__	100%	__	10 000	__	48
CH_4_: 30 H_2_: 30	30 Torr							
CH_4_: 0.5–3	AP	1035	5–20	100%	6 × 6 cm^2^ square arrays	__	__	37
CH_4_: 0.5 H_2_: 10 Ar: 500	AP	1010	10–30	__	__	__	__	49
CH_4_ H_2_	100 Torr	900–930	5–120	100%	5.08 cm	10 620 (max. value)	__	33
CH_4_: 4.6 H_2_: 100 Ar: 200	AP	910	90	∼90%	Hundred nanometers	__	__	61
CH_4_: 0.7 H_2_: 40 Ar: 220	AP	916	105	98%	400–500 nm	__	__	62
CH_4_ H_2_ Ar	AP	916	600	100%	4 inches	>20 000 (over 70% area)	__	63
C_3_H_6_	1.0 × 10^−6^ mbar	600–680	5	100%	__	__	__	68
CH_4_: 50 H_2_: 10 Ar: 300	AP	750	60	100%	6 inches	9700 (RT)	__	64
CH_4_ (1% CH_4_ diluted in Ar)	AP	1075	5	≥98%	__	11 129 (max.) 5273 (average)	650	70

**Table 2:** Dealing process of substrates for the growth of single-crystal graphene

Substrate	Methods	Annealing				Ref.
		Gas flow (sccm)	Press.	Temp. (°C)	Time	
Cu (111)	Repeated chemical mechanical polish and anneal	Ar: 1000 H_2_: 500	AP	1075	2 h	18
	Temperature-gradient-driven annealing technique	Ar: 500	AP	1030	__	36
	Anneal for a long time	Ar H_2_: 100	26 Torr	1030	12 h	43
	Heat Cu foil at 1085 °C in 60 min and anneal it under H_2_ atmosphere on Mo foil	Ar: 300 H_2_: 100	AP	1085	3 min	44
	Make copper foil into a hole-pocket shape	Ar: 500 H_2_: 25	AP	1050	40 min	45
	Pre-introduce Cu oxide layer (heat at 200–350 °C for 10–30 min in air) and anneal	Ar: 1000	AP	1060	70 min	47
	Anneal	Ar: 50 H_2_: 50	760 Torr	1060	18 h	48
Cu (100)	Monocrystallize Cu foil *via* oxygen chemisorption-induced reconstruction	__	1 Pa	1035	0.5–1 h	37
High-index Cu facets	(i) Pre-oxidation polycrystalline Cu foil in air at 150–650 °C for 1–4 h	Ar: 800 H_2_: 50	AP	1020	3–10 h	49
	(ii) Anneal					
Ge (110)	Epitaxially grow H-terminated Ge surface on the Si substrate by introducing GeH_4_ gas as a precursor	__	__	600	30 min	33
Ge (001)	Create the Ge (001) surface with a 9° miscut and anneal	H_2_: 100 Ar: 200	AP	910	30 min	61
	Create Ge (001) surface with a 12° miscut	__	AP	__	__	62
	Create Ge (001) surface with a 15° miscut	H_2_, Ar	__	916	1 h	63
Ni (111)/MgO	(i) Deposit the 50 nm thick Ni film on MgO at 300 °C	__	UHV	700–800	__	68
	(ii) Deposit the 100 nm thick Ni film on top of the first layer at 600 °C					
	(iii) Anneal					
	(iv) Clean the Ni film with Ar^+^ sputtering and anneal					
Cu/Ni (111)	(i) Sputter the Cu/Ni alloy on sapphire with the Cu_85_Ni_15_ alloy target	Ar: 300 H_2_: 20	AP	750	1 h	64
	(ii) Anneal					
	(i) Anneal Cu foil	(i) Ar: 10 H_2_: 10	AP	1050	12 h	70
	(ii) Deposit the Ni layer on the Cu foil and anneal	(ii) Ar: 20 H_2_: 20	AP	1050	4–6 h	

### 3.2 Hexagonal boron nitride

Hexagonal boron nitride (*h*-BN), whose nickname is “white graphene”, has a closer lattice resemblance with graphene. Unlike graphene, it includes B and N atoms alternating alignment in a hexagonal plane instead of only C atoms. Meanwhile, *h*-BN is a superior insulator that offers great promise for electronic applications. Since low-dimensional BN isolation was first reported,^71^ many scientists began to fabricate thin sheets, and even monolayer *h*-BN. Up to date, *h*-BN has shown many excellent properties, such as high transparency, thermal conductivity and superior elastic. The following part will be the growth of the *h*-BN single crystal on different substrates.

#### 3.2.1 Solid Cu catalyst

**Fig. 9:**
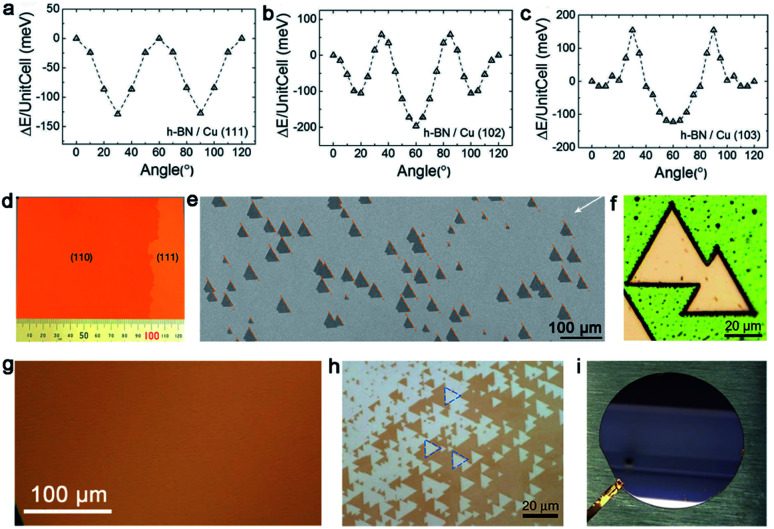
Growth of the single-crystal *h*-BN film on the Cu substrate. (a–c) DFT calculations of the stacking energy with different stacking angles on Cu (111), Cu (102) and Cu (103), respectively. Reproduced with permission.^75^ Copyright 2016, Wiley-VCH. (d) Optical images of the single-crystal Cu (110) surface. (e) Unidirectionally aligned *h*-BN domains on the Cu (110) substrate. (f) No GBs lines in the *h*-BN domains after UV oxidation for 30 min. (d–f) Reproduced with permission.^34^ Copyright 2019, Springer Nature. (g) Optical image of the continuous Cu (111) film without twinned grains. (h) Identical orientation of the *h*-BN flakes on the Cu (111) film. (i) Two inches single-crystal *h*-BN film on the Cu (111)/sapphire substrate. (g–i) Reproduced with permission.^35^ Copyright 2020, Springer Nature.

In general, the precursors of growing *h*-BN range from gas to liquid to solid, such as diborane,^72^ ammonia, borazine,^73,74^ ammonia borane and others. Among them, solid ammonia borane was the most popular precursor in most cases. Compared to graphene, *h*-BN has a lower symmetry, which is not suitable for the growth on the Cu (111) surface with a *C*_6v_ symmetry. As a consequence, an ideal substrate for *h*-BN needs to have a low symmetry, such as *C*_3v_, *C*_3_, *σ*_v_ or *C*_1_.^34^ An article also verified this problem.^75^ They reported that adjacent *h*-BN domains growing on the Cu (111) facet have opposite orientations in sharp comparison with the behaviors of the *h*-BN domains with unified orientation growing on Cu (102) and Cu (103). DFT calculations exhibited the relationship between the orientations of *h*-BN nuclei and the corresponding stacking energy on different Cu facets. There was only a single minimum value at 60° for Cu (102) and Cu (103) facets, whereas two minimum values at 30° and 90° for the Cu (111) facet (Fig. 9a–c). To solve this problem, scientists expounded a brilliant method.^34^ They broke the surface symmetry of the original substrates by designing steps to further achieve the production of a large-area even industrial level single-crystal *h*-BN film. First, they annealed commercial copper at a high temperature (1060 °C), and then it was long-time annealed at 1040 °C until a single-crystal Cu (110) was prepared (Fig. 9d). Taking it as a growth substrate, over 99.5% *h*-BN domains aligned along the 〈211〉 direction (Fig. 9e). Upon more extended growing time, domains would coalesce into a continuous single-crystal *h*-BN film with areas of 10 × 10 cm^2^. Ultraviolet-light oxidization was carried out to further demonstrate its quality. As a result, there were no boundaries on the film (Fig. 9f).

However, it does not mean Cu (111) is of no significance. Meanwhile, a previous report showed that using the Cu/sapphire substrate can achieve the growth of monolayer *h*-BN. It is a concern that the film is a polycrystal with GBs in most cases.^76^ Despite such dilemmas, a recent academic breakthrough was made by the Li group.^35^ As reported in *Nature*, they sputtered a polycrystalline Cu film on *c*-plane sapphire and annealed the system at 1050 °C to eliminate the twin grains on the Cu (111) surface (Fig. 9g), which was essential for the growth of the mono-orientated *h*-BN flakes. Meanwhile, the Cu (111) thin film annealed at 1000 °C had two in-plane orientations, which is consistent with previous reports.^76,77^ Under the guidance of steps on the Cu (111) surface, a unified orientation for the *h*-BN flakes was achieved (Fig. 9h), and was followed by the joining of these flakes to obtain a one-inch single-crystal *h*-BN film, and even a large area of two-inch wafer (Fig. 9i). This cost-effective method allowing for the growth of high-quality *h*-BN films further fills the gaps between laboratory and industry.

#### 3.2.2 Liquid Cu catalyst

**Fig. 10:**
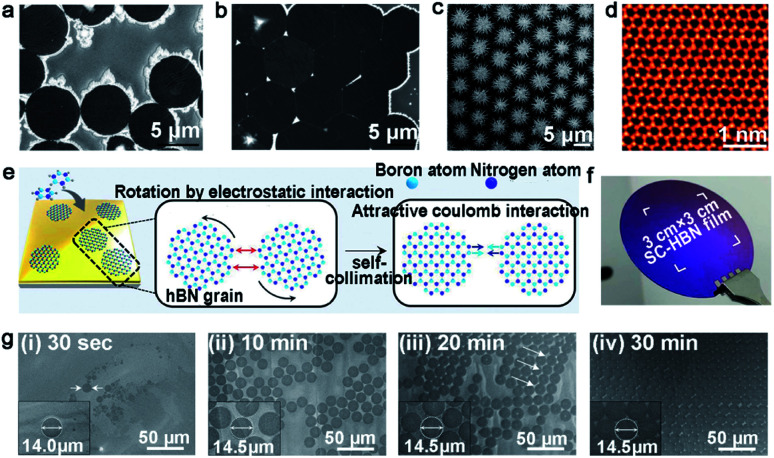
Self-assembled *h*-BN domains on liquid metal. (a and b) SEM images of *h*-BN SASCA with circular edges. Reproduced with permission.^78^ Copyright 2015, WILEY-VCH. (c) SEM image of the hierarchical *h*-BN super-ordered structure. (d) STEM image of the monolayer *h*-BN film exfoliated from the first layer. (c and d) Reproduced with permission.^79^ Copyright 2018, WILEY-VCH. (e) Schematic illustration of growing the *h*-BN film on Au. (f) Photograph of the single-crystal *h*-BN film. (g) The growing process of the *h*-BN film at different times. (e–g) Reproduced with permission.^80^ Copyright 2018, The Authors, published by American Association for the Advancement of Science (AAAS).

Liquid Cu surface with a quasi-atomic smooth surface and amorphous atomic structure has achieved the growth of the *h*-BN film with less defects. The Fu group achieved the growth of the *h*-BN self-aligned single-crystals array (SASCA) on liquid metal for the first time.^78^ They used solid ammonia borane as the precursor and introduced it into a designed set-up, whose inner quartz with one end was closed to eliminate precipitation of the solid precursor. Then, ammonia borane decomposed and *h*-BN was subsequently homogeneously nucleated, growing to cover the whole liquid Cu surface. It should be noted that all of the *h*-BN domains represented a uniform circular shape and accordant spatial orientation due to the isotropic homogeneity of liquid Cu (Fig. 10a and b). Additionally, the extra increment of the precursor would give rise to the growth of a snowflake-like structure. As for their quality, the *h*-BN SASCA electronic device exhibited high insulating property and the *I*–*V* curve showed no current flowing through the devices.

Geng *et al.* developed a method of growing hierarchical *h*-BN super-ordered structures.^79^ Ammonia borane was chosen as the *h*-BN precursor, which is the same as that for the Fu group. Two strategies were applied in this experiment to guarantee a stable precursor gas: (1) set precursor powder into a tube with one end sealed; (2) use another tube with two ends open to store the Cu catalyst. They used W or Mo as the substrate of copper, and optimized the growth parameters to control the growth of the hierarchical structure. As the first nucleation occurred, the *h*-BN domains were arrayed into a unified orientation. At the same time, extra nucleation occurred on the center of the *h*-BN domains and transformed into branched structures. With extended time, the first layer would grow into a hierarchical *h*-BN pattern covering the surface entirely (Fig. 10c). A high-quality monocrystalline *h*-BN film could be simply exfoliated from the hierarchical *h*-BN (Fig. 10d). It was surprising that the whole process lasted several minutes, and the size of the hierarchical *h*-BN was up to centimeter level.

#### 3.2.3 Au catalyst

Except for liquid Cu, liquid Au was employed to grow a wafer-scale single-crystal monolayer *h*-BN film.^80^ It is worth noting that the attractive electrostatic interaction between the B and N atoms on liquid Au is the most important prerequisite for this success (Fig. 10e). Under the specified experimental conditions, the circle *h*-BN grains began to grow, and displayed different behaviors varying with time. The circle domains self-collimated towards a single orientation at a growth time of 20 min, and a continuous film without GBs was obtained at 60 min growth time after the joining of the well-aligned *h*-BN domains (Fig. 10g). NLC was coated on the *h*-BN film to prove its single crystalline property. The POM images directly demonstrated the existence of the large-area monocrystalline *h*-BN. As shown in Fig. 10f, the size of the single-crystal *h*-BN film reached up to 3 cm by 3 cm in dimensions, which opened a new horizon for growth and further application of single-crystal 2D materials.

### 3.3 Other 2D materials

TMDs, a kind of layer materials consisting of a transition metal element and chalcogen atoms, have broad applications at present.^81,82^ To the best of our knowledge, TMDs can be divided into semi-conductor and metallic counterparts. The former mainly are MoS_2_, MoSe, WS_2_, and WSe, and the later involves VS_2_, NbS_2_, TaS_2_ and others. Similar to the growth of other 2D materials, the lattice structure and symmetry of the substrate play a decisive part when growing a large-area continuous TMDs film. At present, insulating materials such as sapphire,^83,84^ mica^85^ and Si/SiO_2_ (ref. 86), in view of their perfect lattice matching, are deemed as appropriate substrates for the growth of TMDs. On top of this, conductive materials such as gold foil can be substrates as well.^87,88^ Then, some particularly recent progress in this field will be presented.

**Fig. 11:**
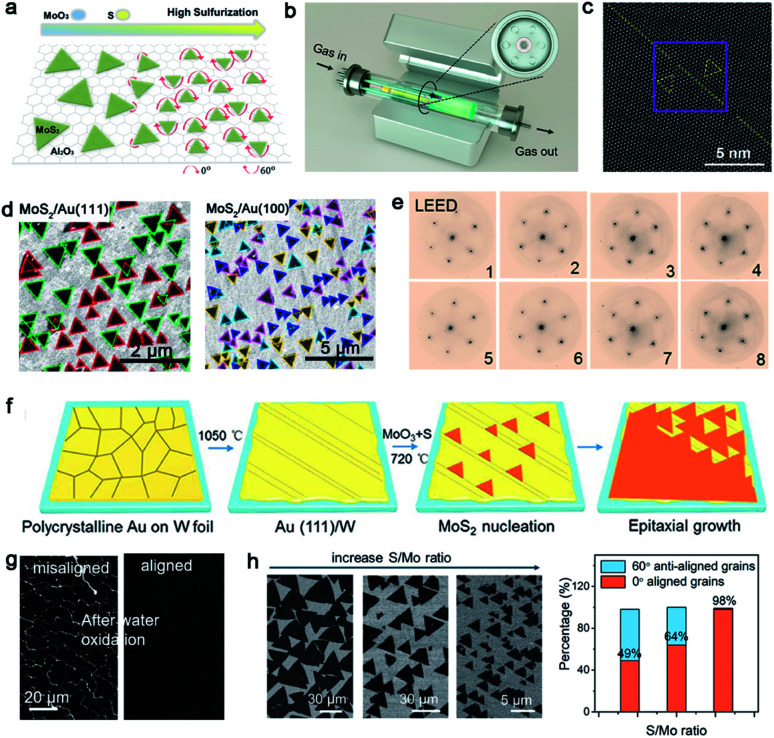
Controllability of orientations by tuning the growth conditions. (a) Schematic showing the directions of the MoS_2_ domains with increasing S precursor. Reproduced with permission.^91^ Copyright 2017, American Chemical Society. (b) Schematic illustration of the experimental setup of the MoS_2_ growth. (c) STEM images showing the MoS_2_ domain boundary. (b and c) Reproduced with permission.^84^ Copyright 2020, American Chemical Society. (d) SEM images of the MoS_2_ domains growing on the Au (111) and Au (100) facets, respectively. Reproduced with permission.^92^ Copyright 2020, IOP Publishing. (e) LEED patterns of 8 regions indicating the single-crystalline nature of the MoS_2_ film. (f) Schematic illustration showing the preparation of the single-crystal Au (111) film and the growth process of MoS_2_. (g) SEM images of the MoS_2_ film after water oxidation formed by misaligned domains and aligned domains, respectively. (h) Orientations of MoS_2_ domains at different S/Mo ratios and statistical histograms of the rotation angle of the MoS_2_ domains. (e–h) Reproduced with permission.^87^ Copyright 2020, American Chemical Society.

Molybdenum disulfide (MoS_2_), as one of the most popular materials, has attracted considerable attention.^89^ Broad experimental research studies have concluded that the concentration of O_2_, the growth temperature and the ratio of S/MoO_3_ affect the properties of the as-obtained MoS_2_ as well.^90,91^ A large-area high-quality film with less GBs is an ultimate goal. In 2017, Li *et al.* found that tuning the ratio of S/MoO_3_ could achieve the control of the MoS_2_ domains' orientation.^91^ A relatively higher S/MoS_3_ ratio was needed at the initial stage (Fig. 11a) because the size of the nucleated grains was small under this condition, and they were prone to rotate spontaneously to reach the energetically preferred stage. That means the domains would regularly align along certain directions, rather than be distributed randomly. Meanwhile, the nucleated seeds would be grown at 750 °C and gratifyingly, the lateral size of the domains would be enlarged when the temperature increased to 800 °C. In order to obtain a large-area homogeneous MoS_2_ film, Zhang *et al.* explored the multisource design for the precursor supply.^84^ As shown in Fig. 11b, MoO_3_ sources were placed in six minitubes surrounding the S source, which was loaded in the center tube. The unusual device guaranteed the uniform growth of 4 inches MoS_2_ film. Besides, the other growth conditions like the O_2_ flux and growth temperature were optimized as well. They confirmed a direct trend of less domains nucleated upon increasing the concentration of O_2_. Choosing an oxygen flow rate of 10 sccm and growth temperature at 930 °C, the domains exhibited only two orientations, namely 0° and 60°. Indeed, there is still the presence of mirror twin boundaries owing to the lattice matched between MoS_2_ and the sapphire substrate (Fig. 11c).

Whereas, why the domains prefer to present two directions and how to change it into one direction are hurdles to solve for higher-quality 2D materials. Recently, Pan *et al.* systematically studied the directions of the MoS_2_ domains on Au foil with different Miller index planes.^92^ With the aid of the STM technique, they proposed that the edges of the MoS_2_ domains preferred to align along the close-packed directions of the Au surface. The bunched step, formed along the close-packed directions as well, further facilitated the preferential nucleation of MoS_2_. The results showed that the Au (111) facet exhibited orientations of [0−11], [−101] and [−110], as well as Au (100) facet with the [011] and [01−1] directions of the step edges. Thus, as shown in Fig. 11d, the MoS_2_ domains exhibit two preferred orientations on the Au (111) surface and four dominated directions on the Au (110) facet. As previous reported, the Liu group achieved the growth of the monocrystalline *h*-BN film on the vicinal Cu (110) surface. They ensured that 99 percent of the *h*-BN domains were attached to the Cu 〈211〉 direction by creating step edges.^34^ On the basis of this strategy, the Zhang group developed a technique of using a vicinal Au (111) thin film to grow a wafer-scale single-crystal MoS_2_ film.^87^ They heated the commercial Au foil at a high temperature of 1050 °C to reach its molten state. Then, the molten liquid Au re-solidified during the cooling procedure and formed the monocrystalline Au (111) facet spontaneously. Thereinto, the most minimum surface energy of Au (111) provided the driving force in this process (Fig. 11f). As for the growth of MoS_2_, MoO_3_ and S were chosen as precursors *via* APCVD method. SEM images demonstrated the well-defined orientation of the as-grown MoS_2_ domains, and individual domains would merge into a continuous film with extended time. Afterwards, 8 representative regions on the as-obtained film were chosen randomly to conduct the LEED measurement to prove the quality of the sample (Fig. 11e). Gratifyingly, all of them exhibited nearly identical lattice orientations, which was an indication of the high crystal quality. Meanwhile, the continuous film was free of the etched boundary lines with respect to the misaligned domains after the water oxidation process (Fig. 11g). With a combination of the experimental phenomenon and theoretical calculation, they proposed a step-guided mechanism, namely the edges of the MoS_2_ domains docked with steps on the Au (111) surface. As evidenced by the LEED patterns, the zigzag edges of MoS_2_ coincided with the 〈110〉 direction of Au. The other particular point was that utilizing a high ratio of Mo/S could be conductive to the unidirectional alignment of the domains. As shown in Fig. 11h, the growth of the 60° rotated domains was gradually suppressed with the increase of the Mo/S ratio. Eventually, the percentage of 0° aligned domains would reach up to 98% when the ratio of Mo/S was up to 3 : 1. The strategy for eliminating the twin GBs of MoS_2_ shed light on the growth of the monocrystal TMDs.

**Fig. 12:**
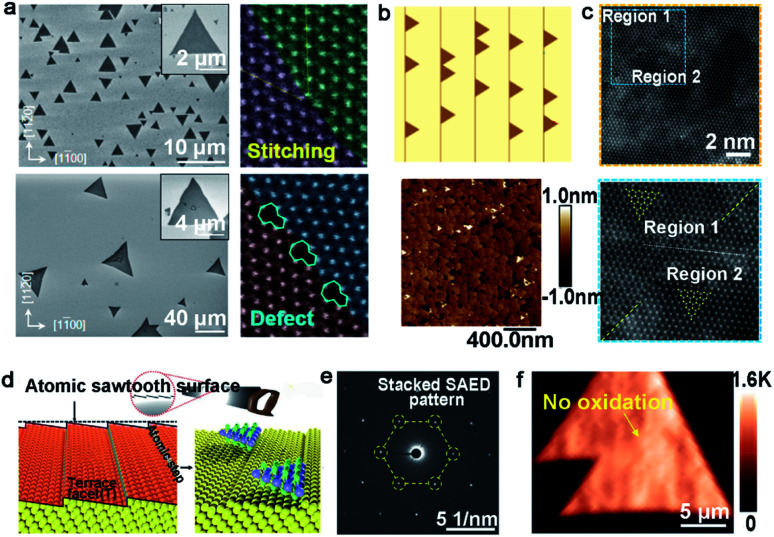
Growth process of the high-quality WS_2_ domains. (a) The orientations of the WS_2_ grains under H_2_ (top) and Ar (bottom) atmospheres, respectively. ADF-STEM images of GBs of the as-grown WS_2_. Reproduced with permission.^94^ Copyright 2017, American Chemical Society. (b) WS_2_ domains aligning along the parallel steps and AFM micrographs of the well-aligned WS_2_ domains on the *c*-plane sapphire. (c) High-resolution atomic images proving the region without inversion domain boundary. (b and c) Reproduced with permission.^95^ Copyright 2021, American Chemical Society. (d) Schematic illustration of the atomic sawtooth Au surface. WS_2_ domains grow along the step edges of the Au surface. (e) SAED pattern indicating the coherent lattice orientation of the WS_2_ film. (f) Raman intensity mapping exhibiting no prominent oxidized GB lines between the aligned WS_2_ domains. (d–f) Reproduced with permission.^96^ Copyright 2021, Wiley-VCH.

Tungsten disulfide (WS_2_), as another semiconductor in the TMDs family, has been regarded as an excellent material for electronic applications.^93^ WO_3_ and S are apt to be chosen as precursors, in addition to Ar as the carrier gas for the growth of WS_2_. In a recent study reported by the Ago group,^94^ the extra introduction of H_2_ at high concentrations (>40%) was used to assist the controllability of the WS_2_ domain orientation. As seen in Fig. 12a, the orientations of the WS_2_ domains were distributed randomly in the case of pure Ar gas. In contrast, there were well-aligned domains in just two orientations under H_2_–Ar mixed gas (H_2_: 50%) conditions. When the neighboring WS_2_ grains had the same alignment, there was a perfect joining without GBs. By means of DFT calculations and water contact angle measurements, they speculated that the existence of H_2_ made the sapphire change into an Al-rich surface. The fact strengthened the interaction between sapphire and WS_2_ grains, which directly affected the alignment of the domains. It should be noted that the alignment of the WS_2_ domains was along the lattice orientation, rather than the step edges.

However, domains grown at the edges of the steps is a common method to control the directions. Furthermore, ensuring regular distributed steps on the substrate is a prerequisite for this achievement. As for sapphire, when the temperature is higher than the critical point, the steps of the substrate would be disordered and were not parallel with each other.^95^ To get out of this dilemma, a group adopted mutable temperature at different stages of growing the WS_2_ monolayer film. In detail, the nucleation of WS_2_ was conducted at 850 °C, and this temperature was kept constant for 20 min, followed by tuning to 1000 °C for 10 min for ripening. Eventually, the lateral growth of the WS_2_ domains was carried out at 1000 °C to achieve a continuous film across the 2-inch sapphire wafer. As shown in Fig. 12b, all of the WS_2_ domains were aligned along the parallel steps of sapphire. The in-plane XRD ω-scan results indicated a low value of the in-plane rotational twist. A low-magnification dark-field image also showed that the as-obtained WS_2_ film was without inversion domain boundaries (Fig. 12c). Meanwhile, the electrical quality was evaluated by back-gated WS_2_ FETs, which showed a high carrier mobility of 16 cm^2^ (Vs)^−1^ and a high *I*_ON/OFF_ of ∼10^7^. Very recently, the Kim group reported the growth of monocrystal TMDs on an atomic sawtooth Au surface.^96^ Different from the vicinal Au (111) surface,^87^ the atomic sawtooth Au surface included regularly distributed step edges and flat terraces, which were obtained by solidifying liquid Au on the W substrate (Fig. 12d). For the growth of WS_2_, the precursor for W was spin-coated onto the Au surface, and sulfur precursors were introduced by bubbling system. At the temperature of 800 °C, WS_2_ began to grow and all gains were well-aligned along a certain direction, like Au (211). After growth of 20 min, the domains would seamlessly merge and a centimeter-scale continuous WS_2_ was obtained. Both SAED patterns with a congruent lattice orientation and oxidation test conducted on the WS_2_ film without visible GB lines demonstrated the free GBs of the as-obtained film (Fig. 12e and f). The same results were repeated during the growth of WSe_2_, MoS_2_, heterostructure, and even alloy.

**Fig. 13:**
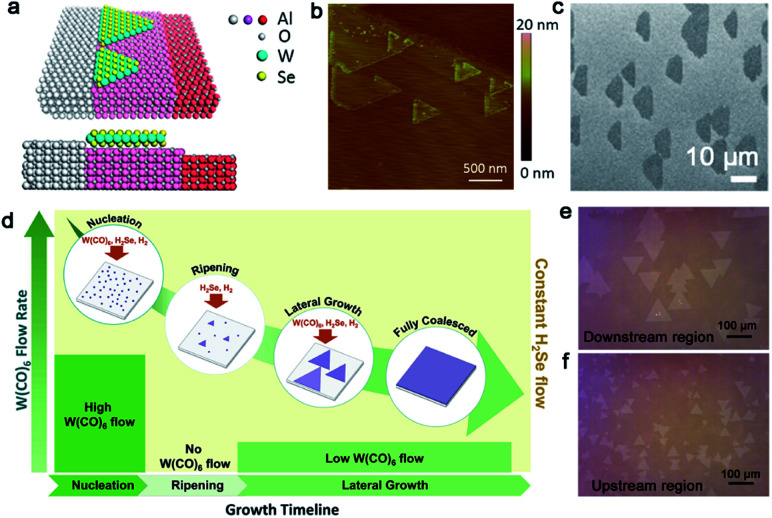
The fabrication process of the WSe_2_ continuous film. (a) Schematic showing the WSe_2_ domains attach to the step edges of sapphire. (b) AFM image of the WSe_2_ domains nucleating at the step edges of sapphire. (c) SEM image of the WSe_2_ domains with a coinciding orientation. (a–c) Reproduced with permission.^83^ Copyright 2015, American Chemical Society. (d) Schematic illustration showing various W(CO)_6_ flow rates at nucleation, ripening, lateral growth, and full coalesced stage of growing WSe_2_. Reproduced with permission.^98^ Copyright 2018, American Chemical Society. (e) OM image of the WSe_2_ flakes grown on the downstream region with two predominant directions. (f) WSe_2_ flakes exhibiting the random distribution on the upstream region. (e and f) Reproduced with permission.^99^ Copyright 2020, Tsinghua University Press and Springer-Verlag GmbH Germany, part of Springer Nature.

In the case of WSe_2_, the CVD method has also been widely employed with Se powders and WO_3_ powders as the precursor. As a member of the TMD family, WSe_2_ has been applied in electronics and valleytronics.^93,97^ Its broad application is based on the high quality and scaled production. In 2015, Chen *et al.* demonstrated the aligned growth of WSe_2_ grains on the *c*-plane sapphire.^83^ By exploration of the appropriate growth conditions, they proved that trapezoid WSe_2_ flakes would regard the step edges of the sapphire substrate as nucleated sites at 950 °C (Fig. 13a), which avoided the nucleation being distributed randomly. It should be noted that the temperature in this process is critical because certain step edges only occurred when the annealing temperature was higher than 950 °C (Fig. 13b and c). The special phenomenon could be ascribed to the high-temperature-triggered surface reconstruction for the sapphire surface. To obtain a large-area homogeneous WSe_2_ film, Zhang *et al.* developed a multistep diffusion-mediated process to control the epitaxial growth process.^98^ They adopted gas precursors like H_2_Se and W(CO)_6_ for the initial growth. By modulating the flow rate of W(CO)_6_ at nucleation, ripening and lateral growth process, an entirely coalesced WSe_2_ film at 1 cm × 1 cm was achieved (Fig. 13d). According to statistical analysis, the WSe_2_ domains showed two preferred directions, namely 0° and 60°, with respect to the [11−20] orientation of sapphire; thus, there existed antiphase GBs. It is indeed a dilemma for scientists to prepare a continuous film with unidirectional aligned domains. Besides, the growth rate of TMDs is still relatively low and the individual domains are usually small. Recently, the Zhou group reported gold-vapor-assisted CVD growing monolayer WSe_2_ with large domain size and rapid growth rate.^99^ There was no doubt that the introduction of the Au vapor catalyzed the chemical reaction dramatically. Meanwhile, they proposed that the growth behaviors of WSe_2_ relied on the specific position of sapphire. When the WSe_2_ domains grew on the downstream region of sapphire, they presented better alignment, large size and monolayer property (Fig. 13e). The average growth rate could reach up to 3.2 μm s^−1^. In contrast, the WSe_2_ flakes grown on the upstream position of the substrate possessed a relatively random distribution of orientations, and the lateral size of each domain was smaller than the downstream position as well (Fig. 13f). The intriguing phenomenon could be ascribed to the concentration of WO_3−*x*_. A lower concentration of precursors led to the growth behaviors of WSe_2_ being under thermodynamic control, where the domains were prone to present good lattice matching with the substrate. Based on these achievements, a more simple and systematic method needed to be explored. How to eliminate the antiphase GBs of TMDs is emergent for practical applications as well.

## 4. Continuous orientated growth mechanism

To the best of our knowledge, the multi-seed strategy is relatively promising for the scaled production of 2D materials. For this method, the dealing and engineering of different metal substrates play a decisive part. Tremendous efforts have been made in the theoretical study, which involves how the metal surface influences the orientations of the 2D single crystals and the detailed behaviors during the coalescence of individual domains. The specific mechanisms vary with different substrates, and we will discuss in this part.

**Fig. 14:**
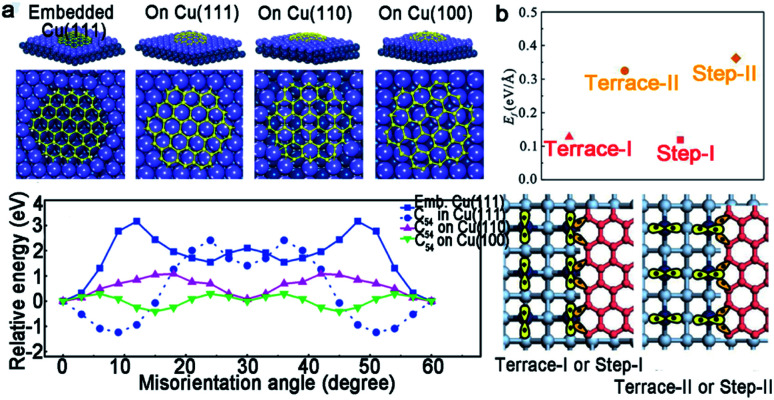
Growth mechanism of the graphene domains with unified orientation on Cu. (a) Schematic of C_54_ embedded Cu (111), on Cu (111), on Cu (110) and Cu (100). DFT calculations of energy difference as a function of misorientation angle (bottom). Reproduced with permission.^18^ Copyright 2014, Wiley-VCH. (b) The formation energy of the graphene edges bonding at different positions. A schematic diagram of the graphene edges bonding with the Ge steps or terrace. Reproduced with permission.^63^ Copyright 2020, Elsevier.

For the Cu (111) substrate, there is a perfect lattice match with graphene. Apart from that, the Lee group established a C_54_ model to understand the orientation dependence on the Cu surface.^18^ C_54_ tended to embed into the Cu (111) surface with a strong confined orientation at *θ* = 0°, as a consequence of the high rotation barrier of about 3 eV (Fig. 14a). In other words, the rotation was unable to occur. However, the graphene growing on the Cu (110) and Cu (100) surfaces had two minimum energies, and a relatively smaller energy difference with the misorientation angle. As a result, the rotation was prone to appear and led to the presence of GBs, which was in accordance with the experimental results. As for the Cu (100) surface, the graphene domains aligning in two orthogonal directions with square shape further exhibited the importance of steps on it.^37^ In fact, graphene domains with coinciding orientation even grew on the Cu facets far from the Cu (111) surface, such as Cu (112) and other high index facets^100^ because the nucleation of graphene has a single lowest energy on these facets.

According to our preceding part of the text, Ge (110) is an ideal substrate for growing single-crystal graphene as a consequence of its asymmetric twofold geometry and the anisotropic nature of the Ge–C covalent bond.^33^ However, as for the Ge (001) facet, the growth of monocrystalline graphene is achieved by the extra creation of the miscut angle.^61–63^ What is more, by increasing the miscut angle, the nucleation orientation of GNRs significantly preferred to be perpendicular to the miscut angle, rather than along with the miscut orientation. As shown in Fig. 14b, the DFT calculations exhibited a different formation energy when the graphene edges were bonded at two kinds of steps or terraces.^63^ As a result, the nucleation unquestionably preferred to occur at Step-I or Terrace-I owing to the lower formation energy instead of another situation, leading to the unidirectional alignment on the vicinal Ge (001) surface.

**Fig. 15:**
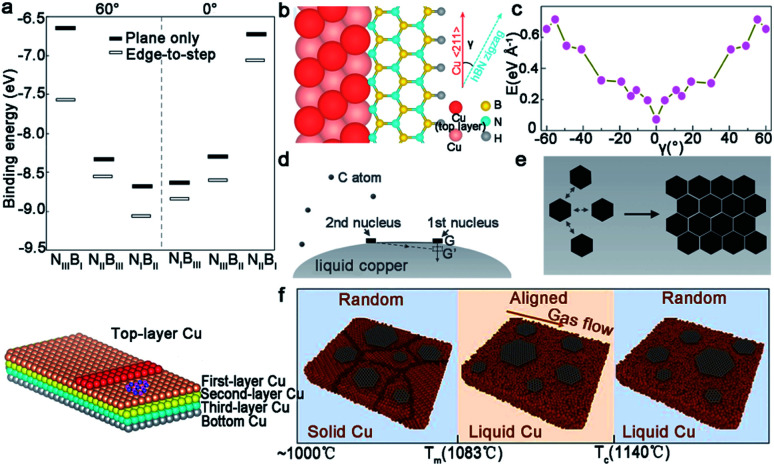
The mechanism of growing single-crystal 2D materials with different catalysts as substrates. (a) Binding energies of different configurations. Schematic exhibiting the edge-docking configuration of *h*-BN (bottom). Reproduced with permission.^35^ Copyright 2020, Springer Nature. (b) Schematic drawing of the zigzag edges bonding with the Cu 〈211〉 step. (c) First-principles DFT calculations showing the formation energy of *h*-BN growing on the Cu (110) surface with steps along Cu 〈211〉. (b and c) Reproduced with permission.^34^ Copyright 2019, Springer Nature. (d and e) The mechanism of the self-aligned graphene arrays on liquid Cu. Reproduced with permission.^53^ Copyright 2014, Wiley-VCH. (f) The evolution of graphene at different temperatures. Reproduced with permission.^102^ Copyright 2019, American Chemical Society.

Switching from graphene to *h*-BN, the Cu (111) and Cu (110) surface will be discussed. Both cases suggest the important of steps in achieving the single-crystallization of the *h*-BN film. For the first case,^35^ a small and rigid B_6_N_7_ molecule was considered to replace *h*-BN to conduct the DFT calculation. The appearance of steps on the Cu (111) surface significantly broadened the gap between the binding energy, which ensured the growth of the mono-orientation (Fig. 15a). The edge-docking mechanism was proclaimed and further emphasized the importance of the step edges, which was the same as the experimental results. For another work, the edge-coupling-guided theory was proposed to explain the behaviors of the *h*-BN domains (Fig. 15b).^34^ The *in situ* observations demonstrated that Cu (110) was vicinal due to the existence of steps, and further characterization demonstrated that all of the step edges were along the 〈211〉 direction. Each single crystal attached to the step edges tightly. Theoretical calculations confirmed that the formation energy of *h*-BN on Cu (110) was only one minimum value (Fig. 15c), which led to the single orientation of the *h*-BN domains on Cu (110). Recently, the Ding group provided deep insight into the relationship between the 2D materials and substrates underneath.^101^ They proposed the importance of the symmetries of substrates and 2D materials, and further concluded that the alignment of the 2D single crystals could be realized only if the symmetry group of the substrate was a subgroup of the target 2D material. It is worth mentioning that the theoretical results provided an extensive scope for growing diverse 2D materials on arbitrary substrates.

When 2D materials grow on the liquid Cu surface, its regular alignment can be realized under some special mechanism. Thereinto, the electrostatic interactions between the liquid metal and the domains play a critical role in the growth of the 2D single-crystal.^56^ The Yu group proposed the surface tension of the liquid Cu to reveal the alignment of the graphene domains.^53^ The whole liquid Cu surface could be seen as a system with a certain arc angle in order to keep balance. When the first nuclei appeared, the balance was disturbed by the additional gravity and needed extra nuclei to come to recover the balance (Fig. 15d). With the extended time, two graphene flakes close to each other followed the surface energy-least principle (Fig. 15e). The same phenomenon repeated until the whole surface was covered by graphene arrays with the unidirectional orientation. On the basis of the recent experimental result,^102^ researchers revealed the effect of temperature and the gas flow's orientation during the alignment of graphene domains on the molten metal surface (Fig. 15f). When the temperature was further below the melting point, the orientations of the graphene domains would depend on the substrate's lattice and the gas flow was unable to trigger the rotation of domains. However, with increasing temperature, the molten Cu gave it a fluid-like property, so that the domains could float and rotate to align with the direction of the gas flow. During this process, the growth temperature has a critical point. If higher than the critical point, the decrease of viscosity for liquid Cu would bring about a strengthened increase in the movement of Cu atoms and the corresponding rotation of graphene. Although a lot of advances have been made, it still needs further study on the mechanism of the growth of 2D materials on liquid metal.

## 5. Applications

Due to the perfect structure of single-crystal 2D materials without GBs, large amounts of excellent properties sparked several interesting and broad applications in the electronics and optics, as well as metal protection. In this section, we will discuss the specific devices one by one based on the high-quality 2D materials.

### 5.1 Electronics

**Fig. 16:**
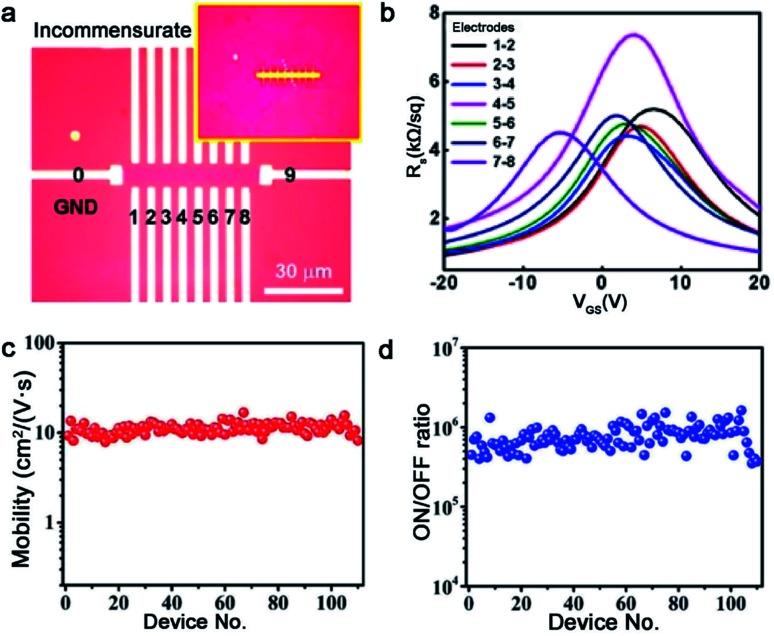
Performance of FETs based on the single-crystal 2D materials. (a) Optical micrographs of the bar-type patterned electrodes with GBs. (b) Sheet resistance between electrodes 0 and 9. (a and b) Reproduced with permission.^18^ Copyright 2015, Wiley-VCH. (c and d) Carrier mobility and ON/OFF ratio of over 110 FETs. Reproduced with permission.^87^ Copyright 2020, American Chemical Society.

At present, FETs are the most basic and important component in the electronics. In recent years, a lot of effort has been made to fabricate high-quality FETs-based 2D materials. Thereinto, high mobility is very important for FETs, although 2D materials with GBs actually show higher electrical resistances due to electronic scattering.^16^ Taking graphene as an example, it exhibits exciting electron mobilities as a consequence of the sp^2^ hybridization of carbon into a 2D honeycomb lattice. So, it is straightforward to fabricate graphene film without GBs, which has a determined effect on the performance of FETs. A previous study confirmed that the polycrystal graphene film fabricated to FETs had a lower field effect mobility of about 3200 cm^2^ V^−1^ s^−1^ and the sheet resistance increased by 60% near GBs (Fig. 16a and b). By comparison, the single-crystal graphene film was about 6100 cm^2^ V^−1^ s^−1^ under the same experimental conditions.^18^ In fact, this value of the single-crystal graphene-based device is still lower than that of the exfoliation flakes. Through further optimizing the parameters of growing and transferring graphene, the electron mobility of FETs can reach up to 23 000 cm^2^ V^−1^ s^−1^,^36^ outperforming by more than that previous reported.

On top of that, *h*-BN also can be used to assist the fabrication of FETs, although it is an electrical insulation. Besides that, *h*-BN also possesses an ultra-flat surface without the presence of dangling bonds and charge traps.^103^ A previous report showed that the loading of *h*-BN on top of the SiO_2_ surface led to the enlarging carrier mobility of graphene-based FETs, which not only prevented the impurities from emerging, but also changed the roughness of the original SiO_2_ substrate.^104^ The carrier mobility of graphene FETs including h-BN was nearly one order of magnitude higher than only SiO_2_. Thereinto, single-crystal *h*-BN is highly desirable for their use as dielectric layers in graphene electronic devices. Gao and co-workers demonstrated that graphene on *h*-BN film exhibited a high-quality electron mobility reaching a level of 15 000 cm^2^ V^−1^ s^−1^, which improved the graphene film properties.^105^ As for TMDs, MoS_2_, as an excellent representative in the family of TMDs, has outstanding electrical properties. MoS_2_ can be used as a conductive channel with HfO_2_ as a gate insulator, as well as exhibited current on/off ratio exceeding 1 × 10^8^ and mobility of ∼200 cm^2^ V^−1^ s^−1^, which was first demonstrated *via* scotch-tape micromechanical cleavage technique.^106^ To date, single-crystal MoS_2_ grown by CVD methods exhibited electrical mobility up to 11.2 cm^2^ V^−1^ s^−1^ and the corresponding on/off ratio of up to 7.7 × 10^5^ (Fig. 16c and d).^87^

Apart from FETs, there are other electrical applications of 2D single-crystal materials. At first, graphene has naturally stimulated the interests in electrodes because of its ultrahigh conductivity. Chen and co-workers showed that a single-crystal graphene film covering on the copper nanowires with a sandwich-structured exhibited great optical and electrical performance in contrast with the polycrystalline graphene-covering copper nanowire counterparts,^10^ which could replace the indium tin oxide electrodes for the fabrication of triboelectric nanogenerators and quantum dot light-emitting diodes.

### 5.2 Optics

The 2D single-crystal materials possess superior optical performance due to their thin layer and perfect lattice structure. Liu and co-workers directly synthesized a 6-inch-scale graphene film on glass *via* oxygen-assisted CVD strategy.^30^ The large-area graphene glass with high transparency was availably fabricated into the transparent electrode for optical filter device. It exhibited excellent performance with a higher transmittance of 92% and lower sheet resistance of 900 Ω sq^−1^. Talking about *h*-BN, different photonic devices based on *h*-BN have been reported in recent years since it possesses remarkable physical properties, such as a wide band gap and high temperature stability. In particular, the deep UV detector based on *h*-BN is widely applied and the single-crystal *h*-BN among them with higher insulating nature further expands the application field of *h*-BN on UV light detection. For instance, deep UV photo detectors fabricated by *h*-BN single-crystal showed barely neglected low dark current,^107^ which was an indicator of the high-quality *h*-BN single-crystal. Under the UV illumination, the device achieved high responsivity up to 5.45 mA W^−1^. Meanwhile, the detectivity of photodetector was about 8.62 × 10^9^ Jones according to corresponding equations. There is no doubt it is a breakthrough for deep UV detection.

### 5.3 Metal protection

**Fig. 17:**
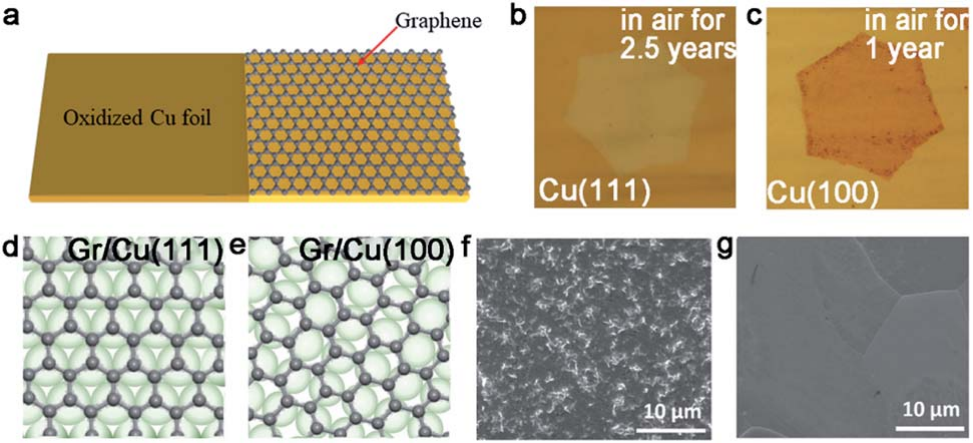
Corrosion of the Cu surface by the coated 2D materials. (a) Schematic showing the color change of the Cu surface without and with the graphene covering, respectively. (b and c) Optical image of the graphene-protected Cu (111) and Cu (100) facets under humid air. (d and e) Schematic showing the lattice structure of the graphene/Cu (111) and graphene/Cu (100) system. (b–e) Reproduced with permission.^106^ Copyright 2017, WILEY-VCH. (f and g) SEM images of the monolayer or multilayer *h*-BN film on the Cu surface immersed in H_2_O_2_ for 11 h. Reproduced with permission.^110^ Copyright 2016, Tsinghua University Press and Springer-Verlag Berlin Heidelberg.

Metal protection has been increasingly necessary for industry and economy. Anti-corrosion coatings used to passivate metal surface worth large-scale market in metal protection field. Owing to the unique structure of the 2D materials, they have been promising candidates in the field of anti-oxidation and anti-corrosion. As for the anti-corrosive coating, there are two important requirements for the effective protection: one is the prohibition of gas penetration onto the metal surface, and another is the elimination of corrosive fluid in-plane diffusion in the interface between the metals and anti-corrosive coating.^108^ Conversely, the metal will be destroyed when the gas or water permeate the metal surface across defects, wrinkles or GBs on the 2D materials.^109^ For graphene, it possesses perfect impermeability to any atoms or molecules under ambient conditions because of the ultra-dense 2D electronic states. Thus, it can better protect the metal from corrosion (Fig. 17a), and should have been widely applied in this field. However, a severe problem should not be overlooked; namely, the high electrical mobility and conductivity of graphene are easy to make itself behave as a cathode to form a galvanic cell with metal underneath, consequently accelerating the corrosion rate.^110^ The single-crystal graphene film thereby becomes a relatively better choice for anti-corrosion application. The Liu group achieved a Cu (111) surface underneath the graphene film that was nicely protected under humid air for 2.5 years with only negligible corrosion (Fig. 17b).^108^ In contrast, the wrinkles on the graphene grown on the Cu (100) surface accelerated the H_2_O diffusion into the interface between them, as well as gave rise to an electrochemical reaction to further enhance the corrosion (Fig. 17c). Thus, the commensurate structure of single-crystal graphene and the Cu (111) surface plays a decisive part in this experiment (Fig. 17d and e). A major difference with graphene is that *h*-BN can provide effective corrosion resistance due to its high electrical insulation nature without the possibility of galvanic cell formation. It is worth mentioning that inevitable defects also degrade the performance of anti-corrosion, and thus single-crystal *h*-BN materials without defects will be more conductive to the practical applications.^111^ Meanwhile, multilayer 2D materials show a better anti-corrosion degree over a long time than the single layer materials. Jiang *et al.*^112^ demonstrated the conclusion by immersing the Cu foil with different thick *h*-BN films in H_2_O_2_. SEM images showed that the copper foil coated with the monolayer *h*-BN film was broken in less than 11 h. Nevertheless, when coated with thicker films, there was almost no degradation (Fig. 17f and g).

## 6. Perspective and prospects

The past few decades have witnessed great progress in growing scaled 2D single crystals by the CVD method, while the engineering of the size, morphology and quality has also been realized. The facts have verified that the multi-seed strategy has huge potentiality in the future development of scaled 2D materials. The merging into a continuous film with well-aligned domains is expected to exhibit huge advantages in comparison with the single-seed strategy that covers the whole substrate by controlling the density of nucleation. The orientation of the nuclei is only determined by the single-crystal structure of substrates, and all of the grains grow at the same time. As a result, this method provides a time-saving chance to industrial production. What is more, the growth rate has been continuously increased in recent years and the size of the 2D single-crystal film even can reach up to the meter-size level.^34,36^ However, the quality and performance of the CVD 2D materials still suffer from other defects compared to mechanically exfoliated materials, such as wrinkles and step bunches. Thus, intensive attention has been contributed to eliminate these defects for high quality 2D materials. For example, the Gao group realized the ultra-flat graphene film without wrinkles by proton-assisted CVD method, extremely improving the intrinsic value of the materials.^113^ Step bunches, which degrade the electrical and mechanical properties, also exist in the process of growth and transfer. Deng *et al.*^114^ found that the roughness of the substrates led to the step bunches. As a result, the ultraflat Cu (111) film could achieve the growth of the ultra-flat graphene film with the roughness down to 0.2 nm. This method could be generally applied to grow other 2D materials, such as *h*-BN. All of the aforementioned advances not only offer a promising future for the development of 2D materials, but also propose the new challenges in this territory. The gap between the laboratory and the factory still exists, and several issues need to be addressed.

At first, fabricating a single-crystal substrate by simple process is essential for growing 2D materials without GBs. Up to now, the majority of substrates need to be annealed at a high temperature in a furnace in order to transform them into a single-crystal structure, which leads to a lot of energy consumption. Meanwhile, the growth temperature of 2D materials is high, generally higher than 700 °C. The high temperature will bring about high energy consumption and cost, thus hampering the development of scaled production. On the one hand, we can sink other precursors to achieve the growth of 2D materials at a lower temperature, such as ethanol or acetylene.^115,116^ Because they have a lower thermal decomposition temperature than methane. To our dismay, the quality of the 2D materials is hard to control at a low temperature. As a result, there are troublesome technological trade-off dilemmas between achieving high-quality and energy-saving. On the other hand, the plasma-enhanced CVD method should be considered for growing 2D materials because it directly happens on the noncatalytic substrates at a lower temperature in comparison with the thermal CVD methods. For example,^6^ a 2D MoS_2_ film was synthesized *via* a single-step PECVD method, using S_8_ and MoCl_5_ as precursors at 500 °C, which was lower than conventional thermal CVD strategy. What is more, there are other advantages for the PECVD method, such as transfer-free and industrial compatibility.^117^

Secondly, improving the yield capacity should be deeply considered during industrialization. At present, there are two main methods to produce 2D materials with high throughput named as batch-to-batch (B2B) and roll-to-roll (R2R). Generally, the area of the 2D materials is limited by the size of the reaction furnace in the B2B process. Increasing the size of the furnace would lead to a challenge for scaled fabrication and economics-saving. To address the issue, the Li group developed a strategy of rolling copper in a vertical direction to realize the growth of the meter level (1.1 m × 0.25 m) graphene film in a 2 inches diameter CVD reactor.^118^ During this process, the Cu foil was rolled into a spiral shape and loaded vertically, and the gas was introduced in a breathing way through repeatedly increasing and decreasing the pressure inside the reactor. By means of this method, the throughput of materials including other 2D materials could be improved with little influence from the size of the CVD reactor. Taking the R2R process into account, the main obstacle is related with the high growth temperature, which results in the energy consumption. With the development of the technique, the R2R microwave plasma (MWP)-CVD achieved the growth of a 294 × 480 mm^2^ area graphene film under 400 °C.^119^ Optimizing the R2R production method and adopting chemical doping, the optical transmittance of a 30-inch graphene film reached up to 97.4% and the sheet resistance was reduced to 120 Ω sq^−1^.^120^ With the development of the technique, the quality and uniformity of the layers are further improved, and the growth mechanism began to be clearly up to date. Polsen *et al.* designed a concentric tube geometry device to grow a continuous graphene film on the metal substrate.^121^ The unique designment ensured the uniform thermal and fluid characteristics of the system. Meanwhile, other process parameters (including growth temperature and annealing period) assisted the fabrication of high-quality graphene. Based on wide-ranging strategies, the Wu group achieved growing 200 × 39 cm^2^ graphene in 15 min by means of the scrolled Cu-graphite structure.^122^ That was adding a graphite paper into the Cu copper, which achieved the high density stacked copper in the small space. The invisibly enlarged device ensured both mass-production and high uniformity of graphene. The production rate could be as high as 520 cm^2^ min^−1^ and the space utilization rate reached up to 0.38 cm^2^ min^−1^. As a result, regardless of the choice of R2R or B2B growth method, both are poor for controlling the layers of 2D materials. Combining the previous conventional CVD method and scaled production method, high-quality and large-scale 2D materials will be promised.

Additionally, to the industrial production, transferring 2D materials into the dielectric substrates for their application needs to be further explored. At present, the complicated PMMA-assisted transfer is the universal method in the laboratory. It is well known that this method is time-consuming and lacks scalability for mass production. Beyond that, it introduces the impurities of polymer residues, as well as defects and cracks towards the perfect 2D material structure. Hence, finding much better methods to transfer the materials need to be continued. Directly growing 2D materials on the dielectric substrates is another possible method. Pang *et al.*^123^ reported the growth of graphene on the Si/SiO_*x*_ substrate with remarkable performance that is highly competitive with graphene grown over other substrates, including Cu. Other dielectric substrates, such as glass,^124^ SiON^125^ and sapphire^126^ can be the template for growing 2D materials as well. It is worth mentioning that Lee and co-workers have achieved directly growing monocrystalline graphene on a single-crystal SiON substrate,^125^ as well as provide a possibility to grow other single-crystal 2D materials on the single-crystal dielectric substrate. Despite the advanced progresses on the dielectric substrate, we still need to surmount the problematic issues, such as the growth rate and the quality of the materials on the arbitrary substrate.

Overall, although we have witnessed great achievements over the last two decades,^127–131^ the growth of scaled single-crystal 2D materials is still challenging. Tremendous efforts still need to be made to bury the giant gap between the laboratory-scale and industry-level in CVD growth of single-crystal 2D materials. Meanwhile, it is essential for further research in fabrication from the consideration of energy-saving, wafer scale, rapid growth and facial transfer. It is hopefully believed that a brighter future for 2D materials will come in the near future.

## Conflicts of interest

There are no conflicts to declare.

## Supplementary Material
